# Phytochemical Profiling and Biological Activities of *Stachys cataonica* Bhattacharjee et Hub.-Mor. Extracts: An LC–MS/MS-Based Approach

**DOI:** 10.3390/molecules31203719

**Published:** 2026-10-16

**Authors:** Erkan Rayaman, Duygu Taşkın, Beyzanur Akduman, Ömer Kılıç, Turgut Taşkın, Hatice Kübra Elçioğlu

**Affiliations:** 1 Department of Pharmaceutical Microbiology, Faculty of Pharmacy, Marmara University, Istanbul 34854, Türkiye; 2 Marmara Pharmacy Drug and Innovative Product Development Unit, Faculty of Pharmacy, Marmara University, Istanbul 34854, Türkiye; turguttaskin@marmara.edu.tr (T.T.); kubra.elcioglu@marmara.edu.tr (H.K.E.); 3 Department of Analytical Chemistry, Faculty of Pharmacy, University of Health Sciences, Istanbul 34668, Türkiye; duygu.taskin@sbu.edu.tr; 4 Department of Pharmacognosy, Faculty of Pharmacy, Marmara University, Istanbul 34854, Türkiye; akduman24@itu.edu.tr; 5 Department of Pharmaceutical Botany, Faculty of Pharmacy, Adıyaman University, Adiyaman 02040, Türkiye; okilic@adiyaman.edu.tr; 6 Department of Pharmacology, Faculty of Pharmacy, Marmara University, Istanbul 34854, Türkiye

**Keywords:** Stachys, *Stachys cataonica*, LC–MS/MS, phytochemical profiling, biological activity

## Abstract

The genus Stachys L. genus is in the Lamiaceae family; comprises many numerous medicinally important taxa traditionally used in Anatolia for nutritional and therapeutic purposes. Many Stachys taxa *Stachys* species have been employed in folk medicine for respiratory, gastrointestinal, inflammatory, metabolic, and skin disorders, as well as wound healing. Despite their recognized ethnopharmacological importance, the chemical composition and biological activities of several endemic Stachys taxa remain insufficiently characterized. *Stachys cataonica* Bhattacharjee et Hub.-Mor. is an endemic species of Türkiye, for which detailed ethnopharmacological information is limited. Therefore, this study aimed to investigate the biological activities and chemical composition of *S. cataonica* extracts and to provide a preliminary characterization of its bioactive compounds. Petroleum ether, chloroform, and methanol extracts of *S. cataonica* were prepared. These extracts were evaluated for antioxidant, antimicrobial, anticholinesterase, and anti-urease activities. An LC–MS/MS-based metabolite profiling approach was subsequently applied to characterize the chemical constituents of the bioactive methanolic extract. Among the tested extracts, the methanolic extract exhibited the strongest DPPH radical-scavenging activity (IC_50_: 0.026 mg/mL), ferric reducing antioxidant power (FRAP: 4.741 mM FeSO_4_ equivalents/mg extract), cupric ion-reducing antioxidant capacity (CUPRAC: 3.758 mM Trolox equivalents/mg extract), anti-urease activity (35.661% inhibition), and antimicrobial activity. Detailed phytochemical analysis revealed that verbascoside was the predominant annotated metabolite in the methanolic extract, accounting for 41.05% of the normalized peak area, followed by caffeic acid (16.71%) and 8,8′-methylenebiscatechin (8.63%), tentatively annotated as a methylene-linked flavan-3-ol dimer. These findings provide preliminary evidence of the bioactive potential of *S. cataonica* and highlight its methanolic extract as a rich source of phenolic and proanthocyanidin-related metabolites. The identified compounds may contribute to the observed biological activities and support further investigation of *S. cataonica* as a potential source of bioactive natural products with pharmacological and biotechnological applications. The metabolite profile obtained in this study expands the phytochemical knowledge of this endemic species and provides a basis for mechanistic and bioactivity-guided studies.

## 1. Introduction

The genus Stachys L., belonging to the family Lamiaceae, comprises more than 360 taxa distributed worldwide and is widely recognized for its ethnomedicinal importance. In Türkiye, the genus is represented by more than 120 taxa, 64 of which are endemic, highlighting the country’s importance as a center of Stachys diversity [1]. Numerous species of this genus have been used in traditional medicine for centuries, particularly in Anatolia, where they serve both nutritional and therapeutic purposes [2].

Traditionally, the aerial parts, leaves, and flowering shoots of *Stachys* species are consumed as herbal infusions or decoctions, often referred to as “mountain tea” in Mediterranean and Anatolian regions [3,4,5]. Their ethnomedicinal applications encompass a broad range of conditions, including respiratory and gastrointestinal disorders, fever, inflammation, rheumatism, asthma, and skin diseases. They have also been used traditionally for wound healing and the management of ulcers, diarrhea, sore throat, and certain hepatic and cardiac complaints [5,6]. In particular, the external application of crushed aerial parts or concentrated decoctions to wounds and inflamed skin is consistent with the traditional use of the common name “woundwort” for certain members of the genus [2,7,8]. Other reported uses include applications as tonics and stomachics, as well as the use of whole plants in herbal preparations traditionally associated with sedative, antispasmodic, diuretic, and emmenagogue effects [5,6,9,10]. Some ethnomedicinal reports also document the use of these plants for serious conditions, including tumors and cancerous ulcers; however, such reports reflect traditional practices rather than established clinical efficacy.

The diversity of traditional applications has stimulated scientific interest in the phytochemical composition and pharmacological properties of *Stachys* species. Previous investigations have reported various biological activities in extracts and isolated constituents, including antioxidant, antibacterial, anti-inflammatory, and other pharmacological effects [5,6,9,10]. The reported antimicrobial properties are particularly relevant in the context of increasing bacterial resistance to conventional antibiotics, which has intensified the search for alternative sources of bioactive compounds [11,12]. Nevertheless, the presence and abundance of bioactive constituents, as well as the magnitude of biological effects, may vary considerably among species and depend on factors such as plant composition, extraction procedures, and experimental conditions.

Despite the extensive ethnomedicinal history of the genus, the phytochemical profiles and biological activities of many endemic Stachys taxa remain insufficiently characterized. This knowledge gap highlights the need for systematic investigations that combine detailed chemical profiling with complementary biological assays. Such an integrated approach can provide a more comprehensive understanding of the relationship between the chemical composition and biological potential of individual species, thereby supporting the scientific evaluation of their traditional uses and identifying candidates for further pharmaceutical and biotechnological research [1,9].

*Stachys cataonica* Bhattacharjee et Hub.-Mor. is an endemic species of Türkiye, occurring mainly on calcareous rocky slopes and limestone cliffs in Malatya and Adıyaman provinces within the Irano-Turanian phytogeographical region [13,14]. The species, along with related *Stachys* taxa, is locally known by vernacular names such as “Dağ Çayı” (mountain tea), “Tüylü Çay,” “Deli Çay,” and “Bodurkarabaş” [3,4]. Although species-specific ethnopharmacological data remain limited, *S. cataonica* has been reported among endemic Stachys taxa used for nutritional and medicinal purposes [5]. Its taxonomic identity is supported by distinctive morphological characteristics, including yellowish corollas, widely spaced verticillasters, triangular-lanceolate calyx teeth, and verrucose, blackish-brown nutlets [14].

Previous studies have investigated the essential oil composition and selected biological properties of *S. cataonica*. Reported volatile constituents include 16-kaurene, α-bisabolol, β-caryophyllene, manoyl oxide, and α-pinene [5,15]. The essential oil has demonstrated cytotoxic activity against human breast cancer (MCF-7) and neuroblastoma (SH-SY5Y) cell lines, while antioxidant and acetylcholinesterase-inhibitory activities have also been reported for the species [5,15]. These findings provide a preliminary basis for further investigation; however, the metabolite profiles of solvent extracts and their relationships with different biological activities remain insufficiently characterized.

Plant-derived secondary metabolites are important sources of compounds with diverse biological activities, including antimicrobial effects. Their investigation has gained particular relevance in the context of increasing antimicrobial resistance, which continues to drive the search for new sources of antimicrobial agents [16,17,18]. Assessing antimicrobial activity alongside antioxidant, anticholinesterase, and anti-urease activities can provide a broader understanding of the biological properties of plant extracts and help identify directions for subsequent pharmacological research.

Liquid chromatography coupled with tandem mass spectrometry (LC–MS/MS) provides an effective approach to investigating complex plant extracts by combining chromatographic separation, mass analysis, and fragmentation patterns. These complementary data facilitate the characterization and tentative annotation of metabolites, supporting the interpretation of phytochemical profiles in relation to observed biological activities. Nevertheless, definitive compound identification may require authentic reference standards or complementary analytical techniques.

Despite previous reports on the essential oil composition and selected biological activities of *S. cataonica*, its solvent-extractable metabolite profile and the biological properties of extracts obtained using solvents of different polarities warrant further investigation. Therefore, this study aimed to evaluate the antioxidant, antimicrobial, anticholinesterase, and anti-urease activities of petroleum ether, chloroform, and methanol extracts of *S. cataonica* and to characterize the phytochemical profile of the bioactive methanol extract using LC–MS/MS.

## 2. Results and Discussion

### 2.1. Antioxidant Activities

The antioxidant activities of petroleum ether, chloroform, and methanol extracts were assessed by FRAP, CUPRAC, and DPPH assays (Table 1).

Significant differences were observed among the extracts depending on solvent polarity. The methanol extract exhibited the strongest reducing power in both FRAP and CUPRAC assays, yielding values of 4.741 ± 0.381 mM FeSO_4_ eq./mg extract and 3.758 ± 0.248 mM Trolox eq./mg extract, respectively. These values were approximately three-fold higher than those obtained for the petroleum ether and chloroform extracts, indicating that polar constituents contributed substantially to the reducing antioxidant capacity.

The DPPH radical scavenging assay further supported the superior antioxidant potential of the methanol extract. Since antioxidant activity in the DPPH assay is inversely proportional to the IC_50_ value, the lowest IC_50_ value indicates the highest radical scavenging capacity. The methanol extract demonstrated the strongest activity with an IC_50_ value of 0.026 ± 0.002 mg/mL, followed by the petroleum ether extract (0.109 ± 0.027 mg/mL), whereas the chloroform extract exhibited the weakest activity (0.462 ± 0.061 mg/mL).

The consistently superior performance of the methanol extract across all antioxidant assays suggests that highly polar phytochemicals are predominantly responsible for the observed antioxidant effects. Phenolic acids, flavonoids, tannins, and other oxygenated secondary metabolites are known to be efficiently extracted by methanol and have been reported as major contributors to antioxidant activity through electron transfer and free radical scavenging mechanisms. The correlation between FRAP, CUPRAC, and DPPH results further supports the presence of potent antioxidant constituents in the methanol fraction.

In contrast, petroleum ether and chloroform extracts showed considerably lower antioxidant activities. The relatively weak performance of these extracts may be attributed to their enrichment in non-polar and moderately polar compounds, which generally possess lower reducing and radical scavenging capacities compared with phenolic-rich polar fractions.

Overall, the methanol extract emerged as the most active antioxidant fraction among the tested extracts, suggesting that polar bioactive compounds constitute the principal antioxidant constituents of the investigated plant material.

### 2.2. Enzyme Inhibitory Activities

The inhibitory activities of the extracts against acetylcholinesterase and urease were evaluated at concentrations of 50 µg/mL and 25 µg/mL, respectively, and the results are presented in Table 2.

The inhibitory effects of the extracts against acetylcholinesterase and urease enzymes are presented in Table 2.

Among the tested extracts, the chloroform extract showed the strongest acetylcholinesterase inhibitory activity (9.014 ± 2.505), followed by the petroleum ether extract (7.292 ± 2.572). The methanol extract exhibited the lowest inhibition against AChE (4.708 ± 2.081).

Similarly, urease inhibition was most pronounced in the methanol extract (35.661 ± 3.531), followed by the chloroform extract (17.207 ± 0.935), whereas the petroleum ether extract displayed relatively weak activity (7.648 ± 2.993).

The marked urease inhibitory activity observed in the methanol extract may indicate the presence of polar bioactive constituents with affinity toward the urease active site. Conversely, the relatively higher AChE inhibition of the chloroform extract suggests that moderately polar secondary metabolites could be responsible for the observed activity.

These findings demonstrate that solvent polarity significantly influences the extraction of biologically active constituents. The methanol extract emerged as the most promising sample regarding antioxidant reducing capacity and urease inhibition, whereas the chloroform extract showed superior acetylcholinesterase inhibitory activity.

Previous investigations of *S. cataonica* have mainly focused on its essential oil and demonstrated its potential antioxidant, acetylcholinesterase (AChE) inhibitory, and cytotoxic activities. The essential oil was evaluated using DPPH, ABTS, and FRAP assays, with the highest antioxidant activity reported in the FRAP assay (IC_50_: 0.612 mg/mL). In addition, the essential oil exhibited noteworthy cytotoxic activity against MCF-7, MRC-5, HeLa, and LNCaP cell lines and showed greater inhibitory activity against AChE. GC–MS analysis identified 16-kaurene (9.93%) and α-bisabolol (9.64%) as major constituents of the essential oil. Thus, the biological potential previously reported for *S. cataonica* has primarily been associated with its volatile constituents [5].

In contrast, the present study investigated different solvent extracts of *S. cataonica* and comparatively evaluated their antioxidant, antimicrobial, anti-urease, and anticholinesterase activities. Among the extracts examined, the methanol extract exhibited the most prominent overall biological activity and was therefore selected for detailed phytochemical characterization by LC–MS/MS. The distinct activity profiles observed among the extracts may be attributed to differences in extraction efficiency and solvent selectivity, which can result in substantial differences in the composition and abundance of bioactive phenolic and other specialized metabolites. The LC–MS/MS analysis of the methanol extract revealed a diverse phytochemical profile dominated by phenylethanoid glycosides and hydroxycinnamic acid derivatives, together with flavonoids and related glycosides and other phenolic constituents. These findings provide a plausible chemical basis for the pronounced biological activity observed for the methanol extract.

The present findings therefore extend the existing knowledge on *S. cataonica* beyond its essential oil by demonstrating substantial biological activity in its solvent-extractable constituents. While previous research established antioxidant, AChE inhibitory, and cytotoxic properties of the essential oil, the present study provides a comparative assessment of different extracts across multiple biological targets and further links the activity of the most active extract with its LC–MS/MS-based phytochemical profile. This integrated approach contributes to a broader understanding of the chemical and biological potential of *S. cataonica*.

### 2.3. Antimicrobial Activity

**Figure 1:**
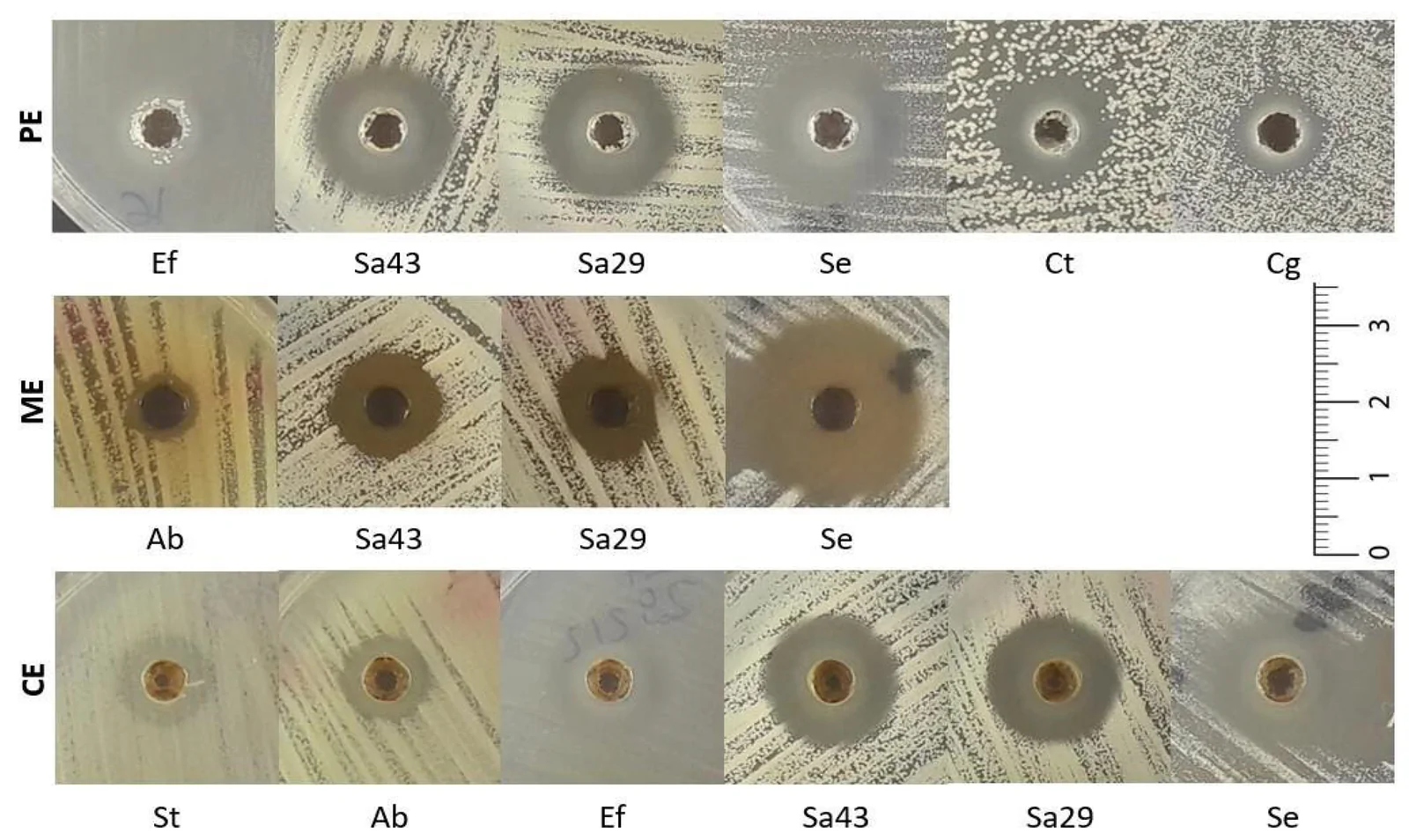
Antimicrobial activity of *S. cataonica* extracts determined by the agar well diffusion method (PE: petroleum ether extract, ME: methanol extract, CE: chloroform extract, Ef: *E*. *faecalis* ATCC 29212, Sa43: *S. aureus* ATCC 43300, Sa29: *S. aureus* ATCC 29213, Se: *S. epidermidis* ATCC 12228, Ct: *C. tropicalis* KUEN 1021, Cg: *C. glabrata* ATCC 90030, Ab: *A. baumannii* ATCC 19606, St: *S. typhimurium* ATCC 14028.

The *S. cataonica* extracts demonstrated varying degrees of antimicrobial activity against the tested microorganisms (Table 3). In general, all extracts were more effective against Gram-positive bacteria, as evidenced by larger inhibition zones and lower MIC values compared to those observed for Gram-negative bacteria. Regarding the yeast strains, only the PE extract exhibited detectable antimicrobial activity (Table 3, Figure 1).

There are limited studies investigating the antimicrobial activity of *S. cataonica*. Therefore, the present study provides valuable data regarding the antimicrobial potential of this plant species. In this study, three different extracts of *S. cataonica* were evaluated against a broad spectrum of microorganisms. The results demonstrated that the extracts exhibited particularly strong antimicrobial activity against Gram-positive bacteria. In addition, the petroleum ether extract was found to possess antifungal activity as well (Table 3). These results indicate that *S. cataonica* extracts have considerable potential for further antimicrobial research, particularly against Gram-positive bacteria.

Among the three metabolites with the highest normalized peak areas in the methanolic extract, verbascoside has been reported to exhibit antifungal activity against *Candida albicans*, *Candida krusei*, *Candida parapsilosis*, *Candida tropicalis*, and *Candida guilliermondii* [19]. In addition, verbascoside has been reported to exhibit antimicrobial activity against *Leishmania infantum*, *Leishmania donovani*, and *Leishmania amazonensis* [20,21]. Regarding caffeic acid, several studies have reported that it exhibits antimicrobial, anti-quorum-sensing, and antibiofilm activities against *S. aureus*. Caffeic acid has also been reported to possess antiviral activity [22,23,24,25]. Although direct information regarding the microbiological activity of 8,8′-methylenebis-catechin is limited, catechins are generally known to possess considerable antimicrobial activity [26]. Taken together, these major constituents may contribute substantially to the antimicrobial activity observed for the methanol extract.

### 2.4. UPLC–QTOF–ESI–MS/MS Characterization of the Most Active Extract

**Figure 2:**
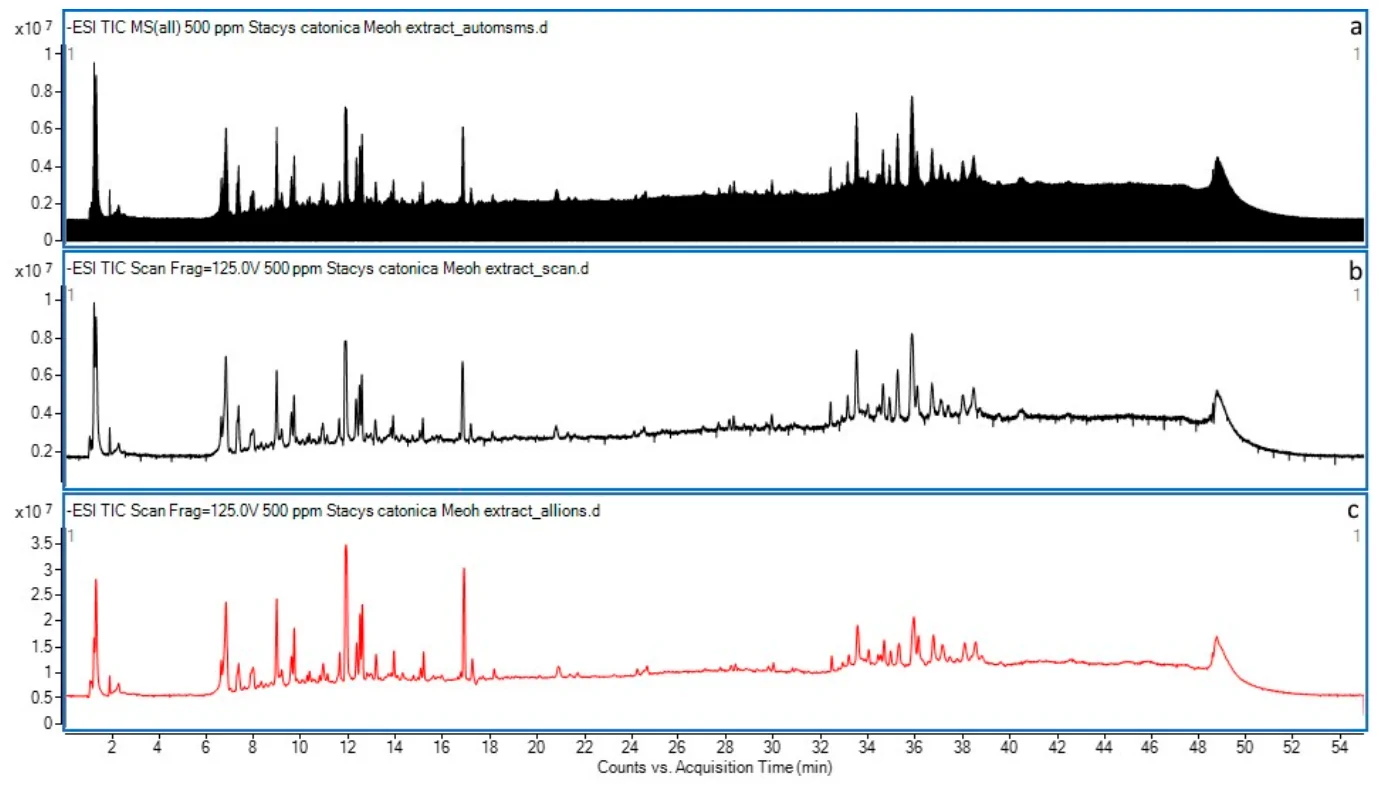
LC–ESI–QTOF–MS/MS chromatographic profiles of the methanol extract of *S. cataonica* acquired in (**a**) AutoMS/MS, (**b**) Scan, and (**c**) All Ions acquisition modes.

**Figure 3:**
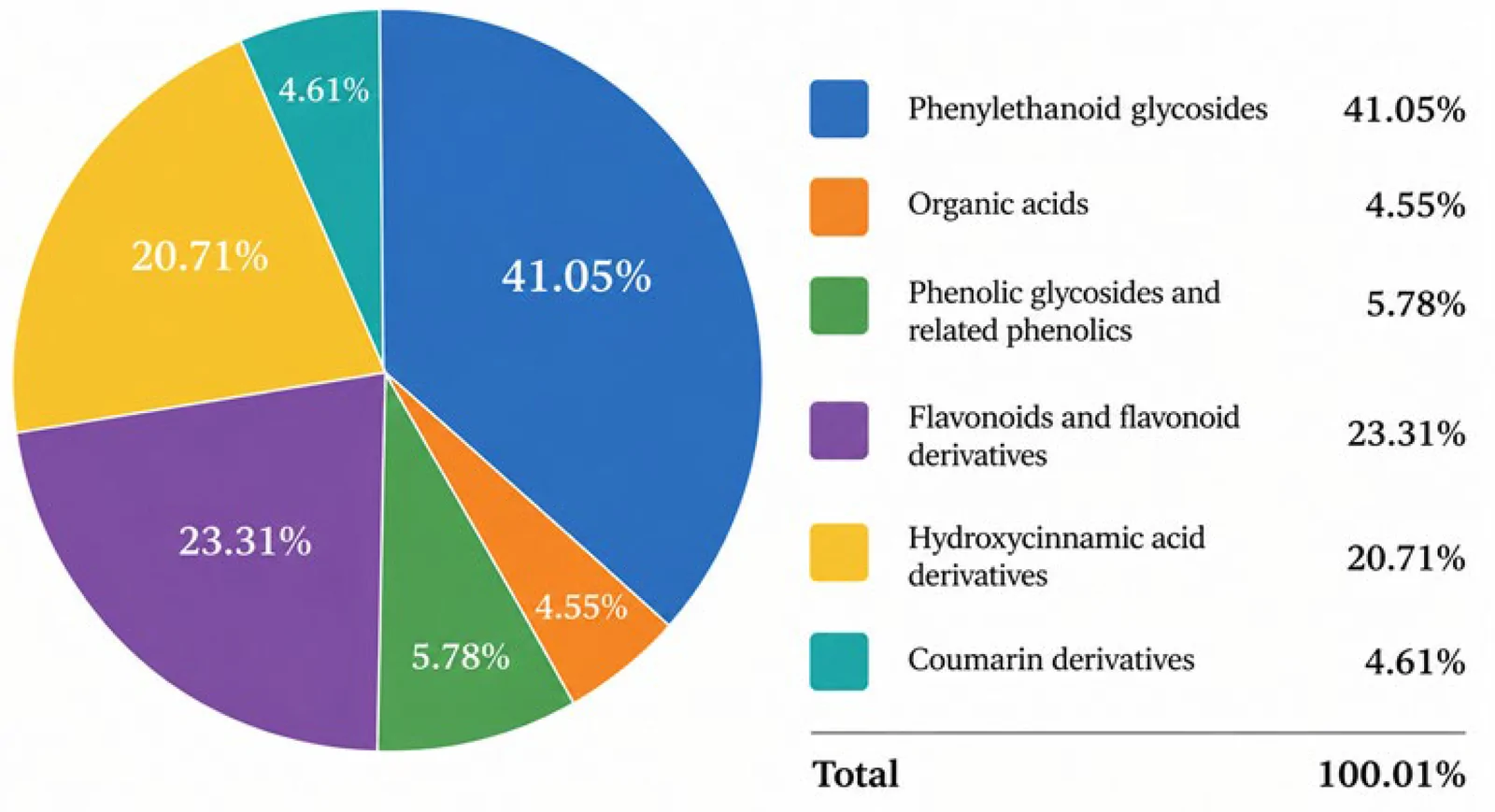
Relative distribution of LC–MS/MS-detected metabolites according to chemical class.

The methanolic extract of *S. cataonica*, which exhibited the strongest overall biological activity among the investigated extracts, was further characterized by UPLC–QTOF–ESI–MS/MS analysis. A total of 18 chromatographic features were tentatively annotated across a retention-time range of 2.566–31.52 min (Table 4, Figure 2 and Figure 3 and Figures S1–S35 (Supplementary Material)). The detected metabolites represented a chemically diverse profile comprising organic acids, phenolic acids and their derivatives, flavan-3-ols, flavonoids, flavonoid glycosides, phenylethanoid glycosides, dihydrochalcone derivatives, anthocyanin-related compounds, sulfur-containing flavonoid derivatives, and coumarin-type constituents. The normalized peak areas of the annotated compounds accounted for 100.01%, with the minor deviation from 100% attributable to rounding. These values represent normalized relative peak areas rather than absolute concentrations, which were not determined using calibration curves.

The earliest-eluting compound was succinic acid (RT 2.566 min), detected as [M − H]^−^ at *m*/*z* 117.0193 and characterized by product ions at *m*/*z* 73 and 55. Its early retention and low molecular mass were consistent with the presence of a highly polar dicarboxylic acid.

In negative electrospray ionization (ESI−) mode, compound 11 was tentatively annotated as 8,8′-methylenebiscatechin based on its mass spectrometric profile and comparison with previously reported fragmentation behavior of catechin-derived compounds. The product-ion spectrum acquired at a collision energy of 41.9 eV showed ions at *m*/*z* 287.9628, 470.0732, and 384.0888, which were considered potentially informative for the fragmentation of the proposed dimeric flavan-3-ol structure. The observed fragmentation pattern was interpreted in the context of cleavage and cross-ring fragmentation pathways reported for catechin derivatives. These findings support the proposed annotation; however, the exact methylene-bridge linkage between the two catechin units remains tentative [27].

Several phenolic acid-related constituents were detected throughout the chromatographic profile. Caffeic acid (RT 9.582 min) was one of the most abundant compounds, representing 16.71% of the normalized peak area. Its [M − H]^−^ ion at *m*/*z* 179.0350 and the characteristic product ion at *m*/*z* 135 supported the annotation. Rosmarinic acid was also detected at RT 17.31 min as a formate adduct, with a relative abundance of 1.20%. In addition, 3,5-dicaffeoyl-4-succinoylquinic acid (RT 9.733 min; 1.18%) was annotated on the basis of its accurate precursor mass and characteristic fragmentation behavior. The detection of this highly oxygenated quinic-acid derivative further demonstrates the structural complexity of the phenolic fraction [28].

Other phenolic glycosides were also observed. Protocatechuic acid 4-O-glucoside at RT 7.519 min produced an [M − H]^−^ ion at *m*/*z* 315.0722 and a characteristic fragment at *m*/*z* 153, consistent with the loss of a hexose residue (162 Da) to generate a protocatechuic-acid-derived product ion. Ethylvanillin glucoside, detected at RT 7.418 min as a formate adduct, was assigned with medium confidence because the observed fragmentation supported a vanilloid-type aromatic structure, whereas the complete glucosylation pattern could not be established solely from the available MS/MS data.

Flavonoid-related constituents were distributed over the early and middle portions of the chromatographic profile. Kaempferol was detected at RT 7.619 min, with the formate-adduct precursor at *m*/*z* 331.0478 and the corresponding kaempferol-derived product ion at *m*/*z* 285. The subsequent fragment ions at *m*/*z* 255, 153, 137, and 121 further supported the flavonol assignment. Vellokaempferol 3-methyl ether (RT 7.871 min) was tentatively assigned based on its accurate precursor mass and flavonoid-type fragmentation pattern, although its structural assignment was considered less secure than that of kaempferol. Similarly, the compound detected at RT 8.005 min was tentatively assigned as 4β-(2,4-dihydroxy-3-methoxyphenyl)fisetinidol. Its [M − H]^−^ precursor at *m*/*z* 411.1084 and product ions at *m*/*z* 375, 300, and 213 were consistent with a fisetinidol-type flavan-3-ol structure; however, the exact positional and stereochemical assignment should be regarded as tentative.

The chromatographic profile also contained several glycosylated flavonoid derivatives. Catechin 3-O-rutinoside (RT 10.50 min) was detected as a formate adduct at *m*/*z* 643.1880, with the formation of the catechin-derived ion at *m*/*z* 289 following cleavage of the rutinosyl moiety. A structurally complex flavonoid glycoside was tentatively annotated as 6-hydroxyluteolin 6,7,3′-trimethyl ether 4′-glucoside at RT 13.17 min. However, the observed product-ion pattern did not independently establish the positions of methylation and glycosylation; therefore, the proposed structure remains tentative [29].

Phenylethanoid glycosides constituted the most prominent chemical class in terms of relative abundance. Verbascoside, detected at RT 12.48 min, accounted for 41.05% of the normalized peak area and represented the predominant annotated metabolite in the methanolic extract. Its [M − H]^−^ precursor ion at *m*/*z* 623.1981 was accompanied by diagnostic product ions at *m*/*z* 461, 315, and 161. The ion at *m*/*z* 461 is consistent with the loss of a caffeoyl-related residue (162 Da), whereas subsequent formation of *m*/*z* 315 is associated with further sugar loss from the *m*/*z* 461 ion. The ion at *m*/*z* 161 is characteristic of the caffeoyl-derived moiety. This fragmentation pattern is consistent with previously reported MS/MS behavior of verbascoside and supports its assignment in the present extract.

The relative-abundance profile showed that verbascoside (41.05%), caffeic acid (16.71%), and 8,8′-methylenebiscatechin (8.63%) were the three most abundant annotated metabolites, together accounting for 66.39% of the total normalized chromatographic peak area.

The relative-abundance profile further indicated that 8,8′-methylenebiscatechin (8.63%) was the third most abundant annotated constituent after verbascoside and caffeic acid. Thus, the three major features—verbascoside (41.05%), caffeic acid (16.71%), and 8,8′-methylenebiscatechin (8.63%)—collectively accounted for approximately two-thirds of the normalized peak area. This distribution indicates that the methanolic extract was particularly enriched in phenylethanoid and phenolic constituents, with additional contributions from flavonoid and other polyphenolic derivatives.

Several less abundant and structurally distinctive metabolites were also tentatively annotated. In negative electrospray ionization (ESI−) mode, the compound was tentatively assigned as 8-C-(p-hydroxybenzyl)kaempferol based on the accurate-mass precursor ion corresponding to the acetate adduct [M + CH_3_COO]^−^ at *m*/*z* 451.1034. The product ions observed at *m*/*z* 218.9542 and 172.8641 were considered potentially informative for the fragmentation behavior of the proposed flavonoid structure. In accordance with established principles of flavonoid fragmentation by tandem mass spectrometry [30] (Cuyckens and Claeys, 2004), these ions may provide additional structural information; however, their specific fragmentation pathways and their ability to establish the C-8 substitution position require further confirmation.

The compound at RT 14.61 min was tentatively assigned as *p*-coumaric acid ethyl ester, whereas the RT 21.28 min feature was annotated as a hydroxy/oxido-coumarin-type compound based on its formate-adduct precursor and the product ion at *m*/*z* 285. Delphinidin 3-O-sambubioside was tentatively detected at RT 18.10 min, although anthocyanin assignments obtained under negative-ion conditions should be interpreted cautiously [31].

Finally, luteolin 7-sulfate-3′-rutinoside at RT 31.52 min was supported by the sequential loss of SO_3_ (80 Da) and the rutinosyl residue, yielding the luteolin-derived ion at *m/z* 285 [32].

Overall, the UPLC–QTOF–ESI–MS/MS profile revealed substantial chemical diversity in the methanolic extract of *S. cataonica*, with phenylethanoid glycosides and phenolic acid derivatives contributing prominently to the normalized peak-area profile. The predominance of verbascoside, together with the relatively high abundances of caffeic acid and 8,8′-methylenebiscatechin, provides a chemically coherent basis for further discussion of the phenolic composition of the most active extract. Nevertheless, because the metabolite annotations were based on accurate mass and MS/MS fragmentation rather than authentic-standard and/or NMR confirmation, structural assignments—particularly for isomeric, highly substituted, and adduct-dependent features—should be regarded as tentative.

LC–MS/MS analysis of the flavonoid-related constituents revealed fragmentation behavior consistent with established gas-phase dissociation pathways of polyphenolic compounds. Neutral losses corresponding to hexosyl (−162 Da), deoxyhexosyl/rhamnosyl (−146 Da), and sulfate (−80 Da) residues were observed in appropriate glycosylated and sulfated flavonoid derivatives, resulting in product ions corresponding to the respective aglycone or aglycone-derived structures. Flavonoid nuclei also exhibited C-ring cleavage and RDA-type fragmentation, generating A- and B-ring-derived product ions whose exact *m*/*z* values were dependent on the substitution pattern of the flavonoid skeleton [33].

In the present dataset, the formation of the kaempferol-derived ion at *m*/*z* 285 from several precursor ions provided useful structural evidence for kaempferol-related derivatives, whereas the formation of the catechin-derived ion at *m*/*z* 289 supported the tentative annotation of 8,8′-methylenebiscatechin. These fragmentation patterns therefore provide complementary evidence for the proposed metabolite classes, while structural isomers cannot be unequivocally distinguished in the absence of authentic standards or orthogonal spectroscopic confirmation.

The relatively high representation of caffeic acid (16.71%) and the detection of rosmarinic acid provide a plausible chemical basis for the antioxidant potential of the methanolic extract, considering the well-established radical-scavenging properties of catechol-containing phenolic compounds. However, the contribution of individual metabolites to the observed antioxidant and enzyme-inhibitory activities cannot be established solely from normalized LC–MS/MS peak areas. Such associations should therefore be considered as chemically plausible rather than causally demonstrated [34].

Comparison with previously investigated Stachys taxa indicates a broadly conserved phytochemical framework comprising phenylethanoid glycosides, hydroxycinnamic acid derivatives, and flavonoids. These metabolite classes have been widely reported across the genus, and verbascoside has been documented in several species, including *Stachys byzantina* K.Koch., *Stachys lanata*, and *S. lavandulifolia* Vahl. [2]. The present analysis further demonstrated that verbascoside was the predominant annotated feature in *S. cataonica*, accounting for 41.05% of the normalized peak area, followed by caffeic acid (16.71%) and 8,8′-methylenebiscatechin (8.63%). The co-occurrence of these phenylethanoid, hydroxycinnamic acid, and condensed flavan-3-ol derivatives indicates a chemically diverse phenolic profile in the methanolic extract. While the relative peak-area distribution suggests a prominent contribution of phenolic metabolites, direct quantitative comparisons with other *Stachys* species should be interpreted cautiously because extraction procedures, analytical platforms, ionization conditions, and reporting units differ among studies. Overall, the present LC–MS/MS data expand the available chemical information on the endemic *S. cataonica* and indicate that verbascoside represents the predominant annotated metabolite based on normalized peak area.

## 3. Materials and Methods

### 3.1. Plant Material and Extraction Procedure

**Figure 4:**
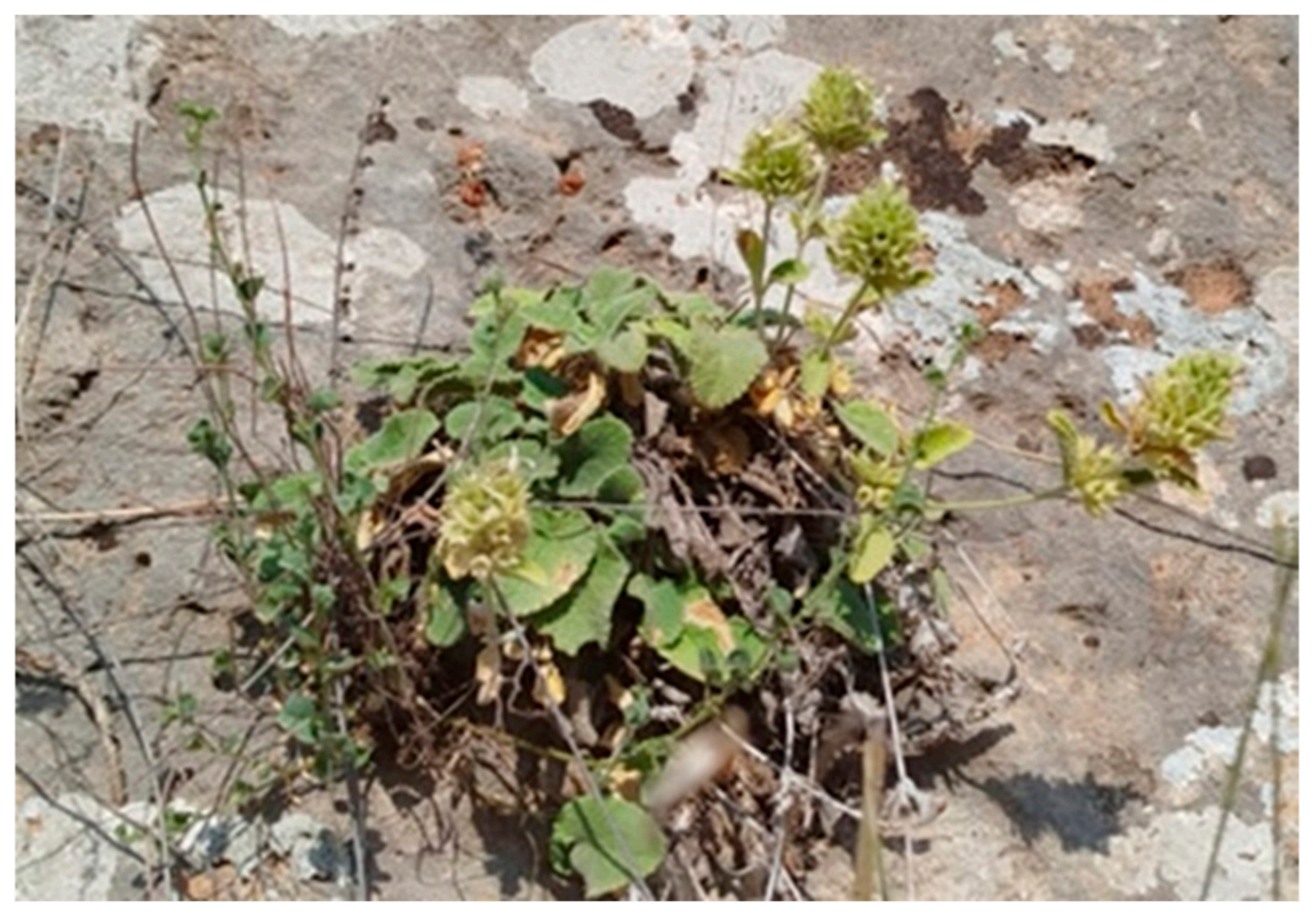
General view of studied plant sample.

*S. cataonica* plant is a perennial woody species. Approximately 100 g of the aerial parts of *S. cataonica* were collected by Dr. Ö. Kılıç in the vicinity of the Levent valley (Akçadağ-Malatya), limestone steppe slopes, and stony-rocky areas at 1190–1220 m on 25 May 2025 (Figure 4). Only the above-ground parts of the plant (not the entire root) were collected in a way that would not harm its habitat or its distribution in future years, and would not negatively affect its sustainability in nature. The plant sample was stored in airy, moisture-free conditions until it was studied. Plant material was dried according to herbarium technique and identified by plant taxonomist Dr. Ö. Kılıç using volume 7 of *Flora of Turkey and East Aegean Islands* [35]. Plant samples are deposited in the Herbarium of Adıyaman University Faculty of Pharmacy and Yıldırımlı Herbarium in Ankara (Türkiye, Voucher No: ÖK 3045).

A total of 100 gr aerial parts of the plant were air-dried at ambient temperature and subsequently ground into a fine powder. Sequential extraction was carried out by maceration using petroleum ether, chloroform, and methanol as extraction solvents. Each extraction step was performed separately, and the resulting mixtures were filtered through Whatman filter paper. The solvents were removed under reduced pressure using a rotary evaporator (Heidolph Hei-VAP Expert, Schwabach, Germany) at temperatures below 50 °C to obtain the corresponding crude extracts. The dried extracts were stored at 4 °C until further phytochemical and biological analyses. The extraction yields were calculated based on the dry weight of the plant material and were found to be 0.67%, 0.65%, and 2.89% for the petroleum ether, chloroform, and methanol extracts, respectively.

### 3.2. Chemicals and Reagents

All solvents and chemicals employed in the present investigation were of analytical or chromatographic grade. Petroleum ether, chloroform, methanol, and other solvents used for extraction and chromatographic analyses were purchased from commercial suppliers (Merck, Darmstadt, Germany) and used without additional purification. Gallic acid, Folin–Ciocalteu reagent, aluminum chloride, and 2,2-diphenyl-1-picrylhydrazyl (DPPH) were utilized for phytochemical and antioxidant analyses. Ferric chloride hexahydrate (FeCl_3_·6H_2_O), 2,4,6-tripyridyl-s-triazine (TPTZ), neocuproine, copper(II) chloride, ammonium acetate, and other reagents required for FRAP and CUPRAC assays were obtained from analytical-grade sources (Sigma-Aldrich, St. Louis, MO, USA). Acetylcholinesterase, urease, their respective substrates, and reference inhibitors (galantamine and thiourea) were purchased from Sigma-Aldrich (St. Louis, MO, USA) and used for enzyme inhibition studies. Microbiological culture media and antimicrobial susceptibility testing reagents were obtained from standard commercial suppliers. Ultrapure water was used in all experimental procedures. LC–MS/MS analyses were carried out using LC–MS-grade methanol, acetonitrile, formic acid, and water supplied by Supelco (Bellefonte, PA, USA) or equivalent chromatographic-grade sources.

### 3.3. Antioxidant Activity Assays

The antioxidant potential of the petroleum ether, chloroform, and methanol extracts was evaluated using ferric reducing antioxidant power (FRAP), cupric ion reducing antioxidant capacity (CUPRAC), and 2,2-diphenyl-1-picrylhydrazyl (DPPH) radical scavenging assays. All measurements were performed in triplicate at a concentration of 0.5 mg/mL. Results were expressed as mean ± standard deviation (SD). A reference blank solution was used in all measurements, and the analyses were performed in triplicate. Results were expressed as mean ± standard deviation (SD).

#### 3.3.1. FRAP Assay

The ferric reducing antioxidant power of the extracts was determined according to a previously reported method. The reduction of Fe^3+^–TPTZ complex to Fe^2+^–TPTZ was monitored spectrophotometrically, and the antioxidant capacity of each extract was calculated from the corresponding absorbance values [36].

#### 3.3.2. CUPRAC Assay

The cupric reducing antioxidant capacity assay was carried out using the Cu(II)-neocuproine reagent system. Following incubation, absorbance was measured spectrophotometrically, and the reducing capacity of the extracts was determined [36].

#### 3.3.3. DPPH Radical Scavenging Assay

The free radical scavenging activity was assessed using the DPPH assay. Extract solutions were mixed with DPPH solutions and incubated in the dark. The decrease in absorbance was recorded, and antioxidant activity was calculated accordingly [36].

### 3.4. Enzyme Inhibitory Activity Assays

The inhibitory effects of the extracts against acetylcholinesterase (AChE) and urease enzymes were evaluated at a concentration of 50 and 25 µg/mL respectively. Galantamine and thiourea were used as positive controls for AChE and urease inhibition assays, respectively. All experiments were conducted in triplicate, and results were expressed as mean ± SD.

#### 3.4.1. Acetylcholinesterase Inhibition Assay

AChE inhibitory activity was determined using Ellman’s colorimetric method. The hydrolysis of acetylthiocholine iodide was monitored spectrophotometrically, and inhibition percentages were calculated relative to the control [37].

#### 3.4.2. Urease Inhibition Assay

Urease inhibitory activity was evaluated using a colorimetric method based on ammonia production from urea hydrolysis. The inhibitory effect of each extract was calculated by comparison with the untreated control [37].

### 3.5. Antimicrobial Activity

#### 3.5.1. Microorganisms and Cultivation

The antimicrobial activity of the extracts was evaluated against major pathogenic microorganisms, including the Gram-negative bacteria *Pseudomonas aeruginosa* ATCC 27853, *Klebsiella pneumoniae* ATCC 4352, *Proteus mirabilis* ATCC 14153, *Salmonella typhimurium* ATCC 14028, *Acinetobacter baumannii* ATCC 19606, and *Escherichia coli* ATCC 25922; the Gram-positive bacteria *Enterococcus faecalis* ATCC 29212, *Staphylococcus aureus* ATCC 43300, *Staphylococcus aureus* ATCC 29213, and *Staphylococcus epidermidis* ATCC 12228; and the yeast strains *Candida albicans* ATCC 90028, *Candida tropicalis* KUEN 1021, and *Candida glabrata* ATCC 90030. Bacterial strains were cultured on Tryptic Soy Agar (TSA) and incubated at 37 °C for 24 h. Yeast strains were cultured on Sabouraud Dextrose Agar (SDA) and incubated at 37 °C for 48 h prior to the experiments.

#### 3.5.2. Agar Well Diffusion Method

The agar well diffusion assay was performed based on European Committee on Antimicrobial Susceptibility Testing (EUCAST) recommendations. Following cultivation, microbial suspensions were prepared in sterile physiological saline (SPS) and adjusted to a turbidity equivalent to 0.5 McFarland for bacteria and 1 McFarland for yeasts. The prepared suspensions were spread onto the surface of Mueller–Hinton Agar (MHA) for bacterial strains and Sabouraud Dextrose Agar (SDA) for yeast strains. Wells with a diameter of 5 mm were then punched into the agar media. Subsequently, 50 µL of each sample was added to the wells. The plates were incubated at 37 °C for 24 h for bacteria and 48 h for yeasts. After incubation, the growth inhibition zones formed around the wells were measured in millimeters (mm) using a digital caliper. All experiments were performed in triplicate. DMSO, SPS, Meropenem (10 µg/well) and amphotericin B (100 µg/well) were used as controls [38,39].

#### 3.5.3. Determination of Minimum Inhibitory Concentration (MIC), Minimum Bactericidal Concentration (MBC), and Minimum Fungicidal Concentration (MFC)

The minimum inhibitory concentration (MIC) and minimum bactericidal/fungicidal concentration (MBC/MFC) of the extracts against microorganisms were determined using the broth microdilution method. The microdilution assay was performed based on EUCAST recommendations. Following cultivation as described in the “Microorganisms and Cultivation” section, microbial suspensions were prepared in sterile physiological saline (SPS) and adjusted to a turbidity equivalent to 0.5 McFarland standard. The suspensions were then diluted to obtain final inoculum concentrations of 5 × 10^5^ CFU/mL for bacteria and 1 × 10^5^–5 × 10^5^ CFU/mL for yeasts. Mueller–Hinton Broth (MHB) was used for bacterial assays, whereas RPMI 1640 medium was used for yeast assays. After dispensing the appropriate culture media into sterile 96-well microplates, two-fold serial dilutions of the extracts were prepared. Subsequently, bacterial or yeast suspensions were added to the corresponding wells, and the microplates were incubated at 37 °C. The incubation (Memmert, Buchenbach, Germany) period was 18–24 h for bacteria and 24 h for yeasts. Following incubation, the lowest extract concentration showing no visible growth for bacteria or causing a ≥90% reduction in yeast growth compared with the growth control was recorded as the MIC. DMSO, meropenem for bacteria, amphotericin B for yeasts, and medium, solvent, and growth controls were included in the experiments. All assays were performed in triplicate [40,41]. For the determination of MBC and MFC values, 10 μL aliquots were taken from wells showing no visible growth in the MIC assay and subculture onto TSA for bacteria and SDA for yeasts. The inoculated plates were incubated at 37 °C for 24 h for bacteria and 24–48 h for yeasts. The lowest extract concentration that resulted in no microbial growth after subculturing was considered the MBC for bacteria and the MFC for yeasts.

### 3.6. UPLC–QTOF–ESI–MS/MS Analysis of the Most Active Extract

The methanol extract was dissolved in LC–MS grade methanol to obtain a stock solution at a concentration of 1000 ppm. Prior to analysis, the solution was diluted to 500 ppm and filtered through a syringe membrane filter (Merck Millipore, Darmstadt, Germany).

Chromatographic and mass spectrometric analyses were carried out using an Agilent 1260 Infinity II UHPLC system coupled to an Agilent 6530 Accurate-Mass QTOF LC/MS instrument equipped with an electrospray ionization (ESI) source (Agilent Technologies, Santa Clara, CA, USA). Separation was performed on a Poroshell 120 EC-C18 column (2.1 × 150 mm, 2.7 µm particle size). The mobile phase consisted of water containing 0.1% formic acid (A) and acetonitrile (B). The chromatographic gradient program and instrumental conditions were applied according to our previously reported method [36]. The flow rate was maintained at 0.4 mL/min, the column temperature was set to 35 °C, and the injection volume was 5 µL.

Mass spectrometric detection was performed in negative ionization mode (ESI−) (Agilent Technologies, Santa Clara, CA, USA). Metabolite annotation was achieved based on accurate mass measurements, retention behavior, isotopic patterns, and characteristic fragmentation pathways obtained from LC–MS/MS analyses. In negative ion mode, compounds were predominantly detected as deprotonated molecular ions ([M − H]^−^), while several metabolites also formed formate adduct ions ([M + HCOO]^−^) due to the presence of formic acid in the aqueous mobile phase.

Data acquisition and processing were performed using MassHunter software packages (version B.12.00, Agilent Technologies). Tentative metabolite annotation was based on accurate mass measurements (mass tolerance below 5 ppm), MS/MS fragmentation patterns, and spectral library matching using MassHunter software. Based on the available spectral evidence, compounds for which the proposed structures were supported by diagnostic fragmentation patterns and library matches were assigned as MSI Level 2 (putatively annotated compounds). As authentic reference standards were not used for experimental confirmation, these annotations were considered tentative. The MS/MS spectra supporting the proposed annotations are provided in the Supplementary Material (Figures S1–S35).

### 3.7. Statistical Analysis

Statistical analyses were performed using GraphPad Prism version 5.0. Differences between groups were analyzed using one-way analysis of variance (ANOVA), followed by Dunnett’s multiple-comparisons test to compare each group with the positive control. A *p*-value of less than 0.05 was considered statistically significant.

## 4. Conclusions

This study presents the first preliminary investigation into the biological activities and LC–MS/MS-based metabolite profile of *S. cataonica*. The methanolic extract exhibited notable antioxidant, antimicrobial, and anti-urease activities, while LC–MS/MS analysis revealed a chemically diverse metabolite profile. Verbascoside, caffeic acid, and 8,8′-methylenebiscatechin were tentatively annotated among the metabolites showing the highest relative chromatographic peak areas. These findings provide a basis for further investigation of the chemical composition and biological activities of *S. cataonica.*

## Figures and Tables

**Table 1:** Antioxidant activities of different *S. cataonica* extracts.

Samples	FRAP (mM FeSO_4_ eq./mg Extract)	CUPRAC (mM Trolox eq./mg Extract)	DPPH IC_50_ (mg/mL)
Petroleum ether extract	1.688 ± 0.402 *	0.589 ± 0.084 *	0.109 ± 0.027 *
Chloroform extract	1.648 ± 0.164 *	0.745 ± 0.011 *	0.462 ± 0.061 *
Methanol extract	4.741 ± 0.381 *	3.758 ± 0.248 *	0.026 ± 0.002 *
Ascorbic acid			0.004 ± 0.001
BHA	5.800 ± 0.104		
BHT		5.683 ± 0.337	

Values are the mean (*n* = 3) ± standard deviation of three parallel measurements; * *p* < 0.05 is evaluated compared to positive control.

**Table 2:** Enzyme inhibitory activities of different *S. cataonica* extracts.

Samples	AChE Inhibitory Activity (%) (50 µg/mL)	Urease Inhibitory Activity (%) (25 µg/mL)
Petroleum ether extract	7.292 ± 2.572 *	7.648 ± 2.993 *
Chloroform extract	9.014 ± 2.505 *	17.207 ± 0.935 *
Methanol extract	4.708 ± 2.081 *	35.661 ± 3.531 *
Thiourea		87.840 ± 0.900
Galantamine	73.805 ± 0.124	

Values are the mean (*n* = 3) ± standard deviation of three parallel measurements; * *p* < 0.05 is evaluated compared to positive control.

**Table 3:** Antimicrobial activity of *S. cataonica* extracts determined by agar well diffusion and microdilution methods.

		Agar Well Diffusion Assay			Microdilution Assay					
		ZD (mm)			MIC (mg/mL)			MBC/MFC (mg/mL)		
		PE	ME	CE	PE	ME	CE	PE	ME	CE
Gram-negative bacteria	*Pseudomonas aeruginosa* ATCC 27853	0	0	0	-	-	-	-	-	-
	*Klebsiella pneumoniae* ATCC 4352	0	0	0	-	-	-	-	-	-
	*Proteus mirabilis* ATCC 14153	0	0	0	-	-	-	-	-	-
	*Salmonella typhimurium* ATCC 14028	0	0	9.82 ± 0.09	-	-	0.39	-	-	0.78
	*Acinetobacter baumannii* ATCC 19606	0	8.13 ± 0.14	11.82 ± 0.12	-	0.78	0.39	-	1.56	0.78
	*Escherichia coli* ATCC 25922	0	0	0	-	-	-	-	-	-
Gram-positive bacteria	*Enterococcus faecalis* ATCC 29212	19.22 ± 0.08	0	11.80 ± 0.12	0.10	-	0.10	0.20	-	0.39
	*Staphylococcus aureus* ATCC 43300	15.52 ± 0.13	13.11 ± 0.09	11.22 ± 0.17	0.05	3.13	0.20	0.1	6.25	0.39
	*Staphylococcus aureus* ATCC 29213	16.82 ± 0.12	11.21 ± 0.14	14.56 ± 0.10	0.02	1.56	0.20	0.05	3.13	0.39
	*Staphylococcus epidermidis* ATCC 12228	12.52 ± 0.10	22.21 ± 0.08	16.01 ± 0.12	0.10	1.56	0.10	0.20	3.13	0.20
Yeasts	*Candida albicans* ATCC 90028	0	0	0	-	-	-	-	-	-
	*Candida tropicalis* KUEN 1021	13.52 ± 0.10	0	0	0.10	-	-	0.20	-	-
	*Candida glabrata* ATCC 90030	11.11 ± 0.08	0	0	0.10	-	-	0.20	-	-

ZD: inhibition zone diameter, MIC: minimum inhibitory concentration, MBC: minimum bactericidal concentration (for bacteria), MFC: minimum fungicidal concentration (for yeast), PE: petroleum ether extract, ME: methanol extract, CE: chloroform extract, -: not done.

**Table 4:** Tentative LC–MS/MS-based metabolite annotation and relative peak-area profiling of detected compounds.

Rt (min)	Compound	Formula	Ion	Exp *m*/*z*	Key Fragments	Area (%)	Diagnostic Interpretation
2.566	Succinic acid	C_4_H_6_O_4_	[M − H]^−^	117.0193	73, 55	4.55	Deprotonated succinic acid; characteristic low-mass organic-acid fragmentation
6.790	8,8′-Methylenebiscatechin	C_31_H_28_O_12_	[M − H]^−^	591.1508	470, 384, 287	8.63	Flavan-3-ol dimer; formation of catechin-derived product ion at *m*/*z* 289 is consistent with cleavage of the methylene-linked dimer
7.418	Ethylvanillin glucoside	C_15_H_20_O_8_	[M + HCOO]^−^	373.1140	332, 240, 123	4.58	Formate adduct; product ion is expected after loss of formic acid, while the observed low-mass ions support a vanilloid-type aromatic moiety; glucoside assignment remains tentative
7.519	Protocatechuic acid 4-O-glucoside	C_13_H_16_O_9_	[M − H]^−^	315.0722	153, 109	1.20	Diagnostic neutral loss of 162 Da (hexose) yielding protocatechuic-acid-derived ion at *m*/*z* 153
7.619	Kaempferol	C_15_H_10_O_6_	[M + HCOO]^−^	331.0478	285, 255, 153, 137, 121	2.55	Formate adduct; loss of formic acid gives kaempferol-derived [M − H]^−^ at *m*/*z* 285, followed by characteristic flavonol/RDA-type fragmentation
7.871	Vellokaempferol 3-methyl ether	C_21_H_18_O_6_	[M + HCOO]^−^	411.1085	375, 300, 213, 169, 59	0.89	O-methylated flavonol; formate-adduct behavior and flavonoid product-ion pattern support the proposed assignment
8.005	4β-(2,4-dihydroxy-3-methoxyphenyl)fisetinidol	C_22_H_20_O_8_	[M − H]^−^	411.1084	375, 300, 213	0.94	Fisetinidol-type flavanol; product-ion pattern is consistent with flavan-3-ol fragmentation, but positional/stereochemical assignment remains tentative
9.196	8-C-p-Hydroxybenzylkaempferol	C_22_H_16_O_7_	[M + CH_3_COO]^−^	451.1035	218, 172, 59	1.55	Putative acetate adduct of 8-C-p-hydroxybenzylkaempferol; formation of the kaempferol-derived ion at *m*/*z* 285 supports the proposed flavonol core
9.582	Caffeic acid	C_9_H_8_O_4_	[M − H]^−^	179.0350	135, 107, 92	16.71	Characteristic decarboxylation (−44 Da) producing *m*/*z* 135, with additional caffeate-related product ions
9.733	3,5-Dicaffeoyl-4-succinoylquinic acid	C_29_H_28_O_15_	[M + HCOO]^−^	661.1410	594, 474, 383,117	1.18	Formate adduct; sequential fragmentation involving caffeoyl/succinoyl-substituted quinic-acid moiety; exact adduct mass agrees with the molecular formula
10.50	Catechin 3-O-rutinoside	C_27_H_34_O_15_	[M + HCOO]^−^	643.1911	513, 440, 283, 137	0.96	Formate adduct; loss of 308 Da corresponding to rutinosyl moiety yields catechin-derived ion at *m*/*z* 289
12.48	Verbascoside	C_29_H_36_O_15_	[M − H]^−^	623.1981	461, 315, 161	41.05	Phenylethanoid glycoside; fragmentation involving caffeoyl and hydroxytyrosol-derived portions is consistent with verbascoside
13.17	6-hydroxyluteolin 6,7,3′-trimethyl ether 4′-glucoside	C_24_H_26_O_12_	[M + HCOO]^−^	551.1431	429, 316, 207, 152, 77	1.72	Formate adduct; exact precursor mass is consistent with the proposed molecular formula, but the observed product-ion pattern does not by itself securely establish the complete substitution/glycosylation pattern
14.61	p-Coumaric acid ethyl ester	C_11_H_12_O_3_	[M + HCOO]^−^	237.0768	219, 173, 136	1.62	Formate adduct of ethyl p-coumarate; product ions are consistent with ester-related fragmentation and the p-coumaroyl moiety
17.31	Rosmarinic acid	C_18_H_16_O_8_	[M + HCOO]^−^	405.0827	359, 197, 161	1.20	Formate adduct; loss of formic acid gives *m*/*z* 359, with subsequent fragmentation consistent with the caffeoyl-derived portions of rosmarinic acid
18.10	Delphinidin 3-O-sambubioside	C_26_H_29_O_16_	[M + HCOO]^−^	643.1492	441, 271, 209, 153, 121	0.92	the elimination of the pentose group from the sambubiose sugar structure (m/z 441) and the characteristic fragmentation products of the anthocyanidin ring (m/z 209)
21.28	7,4,5-Trihydroxy-5′,2′-oxido-4-phenylcoumarin	C_15_H_8_O_6_	[M + HCOO]^−^	329.0313	285.0410	4.61	Formate adduct; loss of formic acid (46 Da) yields *m*/*z* 285.0410 corresponding to the deprotonated molecular ion; supports an oxido-coumarin-type structure
31.52	Luteolin 7-sulfate-3′-rutinoside	C_27_H_30_O_18_S	[M − H]^−^	673.1080	593, 285, 151	5.15	Loss of SO_3_ (80 Da) gives *m*/*z* 593, followed by loss of rutinosyl residue (308 Da) to yield luteolin-derived *m*/*z* 285; additional RDA fragmentation gives *m*/*z* 151

Note: Rt, retention time. *m*/*z* values correspond to deprotonated molecules ([M − H]^−^) or adduct ions formed in negative electrospray ionization (ESI) mode. Area (%) represents the relative peak area normalized to the most intense detected feature (base peak = 100%) in the full-scan LC–ESI–QTOF–MS chromatogram. Compound identification is tentative and is based on accurate mass measurements, isotopic pattern matching, database comparisons, and MS/MS fragmentation data.

## Data Availability

All relevant data are contained within the article and the Supplementary Materials.

## Supplementary Materials

The following supporting information can be downloaded at: https://www.mdpi.com/article/10.3390/molecules31203719/s1, Figures S1–S35. Figure S1. Negative ESI-MS full-scan spectrum of succinic acid; Figure S2. Negative ESI-MS full-scan spectrum of 4β-(2,4-dihydroxy-3-methoxyphenyl)fisetinidol; Figure S3. Negative-ion ESI-MS/MS product-ion spectrum of 4β-(2,4-dihydroxy-3-methoxyphenyl)fisetinidol; Figure S4. Negative ESI-MS full-scan spectrum of vellokaempferol 3-methyl ether; Figure S5. Negative-ion ESI-MS/MS product-ion spectrum of vellokaempferol 3-methyl ether; Figure S6. Negative ESI-MS full-scan spectrum of 8-C-p-hydroxybenzylkaempferol; Figure S7. Negative-ion ESI-MS/MS product-ion spectrum of 8-C-p-hydroxybenzylkaempferol; Figure S8. Negative ESI-MS full-scan spectrum of 3,5-dicaffeoyl-4-succinoylquinic acid; Figure S9. Negative-ion ESI-MS/MS product-ion spectrum of 3,5-dicaffeoyl-4-succinoylquinic acid; Figure S10. Negative ESI-MS full-scan spectrum of verbascoside; Figure S11. Negative-ion ESI-MS/MS product-ion spectrum of verbascoside; Figure S12. Negative ESI-MS full-scan spectrum of 6-hydroxyluteolin 6,7,3′-trimethyl ether 4′-glucoside; Figure S13. Negative-ion ESI-MS/MS product-ion spectrum of 6-hydroxyluteolin 6,7,3′-trimethyl ether 4′-glucoside; Figure S14. Negative ESI-MS full-scan spectrum of catechin 3-O-rutinoside; Figure S15. Negative-ion ESI-MS/MS product-ion spectrum of catechin 3-O-rutinoside; Figure S16. Negative ESI-MS full-scan spectrum of protocatechuic acid 4-O-glucoside; Figure S17. Negative-ion ESI-MS/MS product-ion spectrum of protocatechuic acid 4-O-glucoside; Figure S18. Negative ESI-MS full-scan spectrum of ethylvanillin glucoside; Figure S19. Negative-ion ESI-MS/MS product-ion spectrum of ethylvanillin glucoside; Figure S20. Negative ESI-MS full-scan spectrum of rosmarinic acid; Figure S21. Negative-ion ESI-MS/MS product-ion spectrum of rosmarinic acid; Figure S22. Negative ESI-MS full-scan spectrum of kaempferol; Figure S23. Negative-ion ESI-MS/MS product-ion spectrum of kaempferol; Figure S24. Negative ESI-MS full-scan spectrum of caffeic acid; Figure S25. Negative-ion ESI-MS/MS product-ion spectrum of caffeic acid; Figure S26. Negative ESI-MS full-scan spectrum of delphinidin 3-O-sambubioside; Figure S27. Negative-ion ESI-MS/MS product-ion spectrum of delphinidin 3-O-sambubioside; Figure S28. Negative ESI-MS full-scan spectrum of p-coumaric acid ethyl ester; Figure S29. Negative-ion ESI-MS/MS product-ion spectrum of p-coumaric acid ethyl ester; Figure S30. Negative ESI-MS full-scan spectrum of luteolin 7-sulfate-3′-rutinoside; Figure S31. Negative-ion ESI-MS/MS product-ion spectrum of luteolin 7-sulfate-3′-rutinoside; Figure S32. Negative ESI-MS full-scan spectrum of 8,8′-methylenebiscatechin; Figure S33. Negative-ion ESI-MS/MS product-ion spectrum of 8,8′-methylenebiscatechin; Figure S34. Negative ESI-MS full-scan spectrum of 7,4,5-trihydroxy-5′,2′-oxido-4-phenylcoumarin; Figure S35. Negative-ion ESI-MS/MS product-ion spectrum of 7,4,5-trihydroxy-5′,2′-oxido-4-phenylcoumarin.

## Abbreviations

The following abbreviations are used in this manuscript:

CUPRAC	Cupric ion reducing antioxidant capacity
DPPH	2,2-Diphenyl-1-picrylhydrazyl
IC_50_	Half-maximal inhibitory concentration
BHA	Butylated hydroxyanisole
BHT	Butylated hydroxytoluene
AChE	Acetylcholinesterase
ABTS	2,2′-Azino-bis(3-ethylbenzothiazoline-6-sulfonic acid)
GC–MS	Gas Chromatography–Mass Spectrometry
MIC	Minimum inhibitory concentration
ZD	Inhibition zone diameter
MBC	Minimum bactericidal concentration
MFC	Minimum fungicidal concentration
PE	Petroleum ether extract
ME	Methanol extract
CE	Chloroform extract
UPLC–QTOF–ESI–MS/MS	Ultra-performance liquid chromatography–quadrupole time-of-flight–electrospray ionization–tandem mass spectrometry
MS/MS	Tandem mass spectrometry
NMR	Nuclear magnetic resonance
TPTZ	2,4,6-Tris(2-pyridyl)-s-triazine
EUCAST	European Committee on Antimicrobial Susceptibility Testing
ATCC	American type culture collection
MHA	Mueller–Hinton Agar
SDA	Sabouraud dextrose agar
DMSO	Dimethyl sulfoxide
SPS	Sterile physiological saline
MHB	Mueller–Hinton broth
RPMI 1640	Roswell Park Memorial Institute 1640 medium
