# Supplementary material for: Phytochemical Profiling and Biological Activities of Stachys cataonica Bhattacharjee et Hub.-Mor. Extracts: An LC–MS/MS-Based Approach
Source: Molecules. 2026 Oct 16;31(20):3719. doi: 10.3390/molecules31203719 (PMC13707666; doi:10.3390/molecules31203719)
Supplement: Supplementary file 1 [file molecules-31-03719-s001.zip › molecules-4629231-supplementary.pdf]

Supporting Information

Phytochemical Profiling and Biological Activities of *Stachys cataonica* Bhattacharjee et Hub.-Mor. Extracts: An LC–MS/MS-Based Approach

Erkan Rayaman, Duygu Taşkın, Beyzanur Akduman, Ömer Kılıç, Turgut Taşkın, Hatice Kübra Elçioğlu

Department of Pharmaceutical Microbiology, Faculty of Pharmacy, Marmara University, Istanbul,

Department of Analytical Chemistry, Faculty of Pharmacy, University of Health Sciences, Istanbul, Türkiye

Department of Pharmacognosy, Faculty of Pharmacy, Marmara University, Istanbul, Türkiye

Türkiye

Department of Pharmaceutical Botany, Faculty of Pharmacy, Adıyaman University, Adıyaman 02040, Türkiye

Department of Pharmacology, Faculty of Pharmacy, Marmara University, Istanbul 34854, Türkiye

Marmara Pharmacy Drug and Innovative Product Development Unit, Faculty of Pharmacy, Marmara University, Istanbul 34854, Türkiye

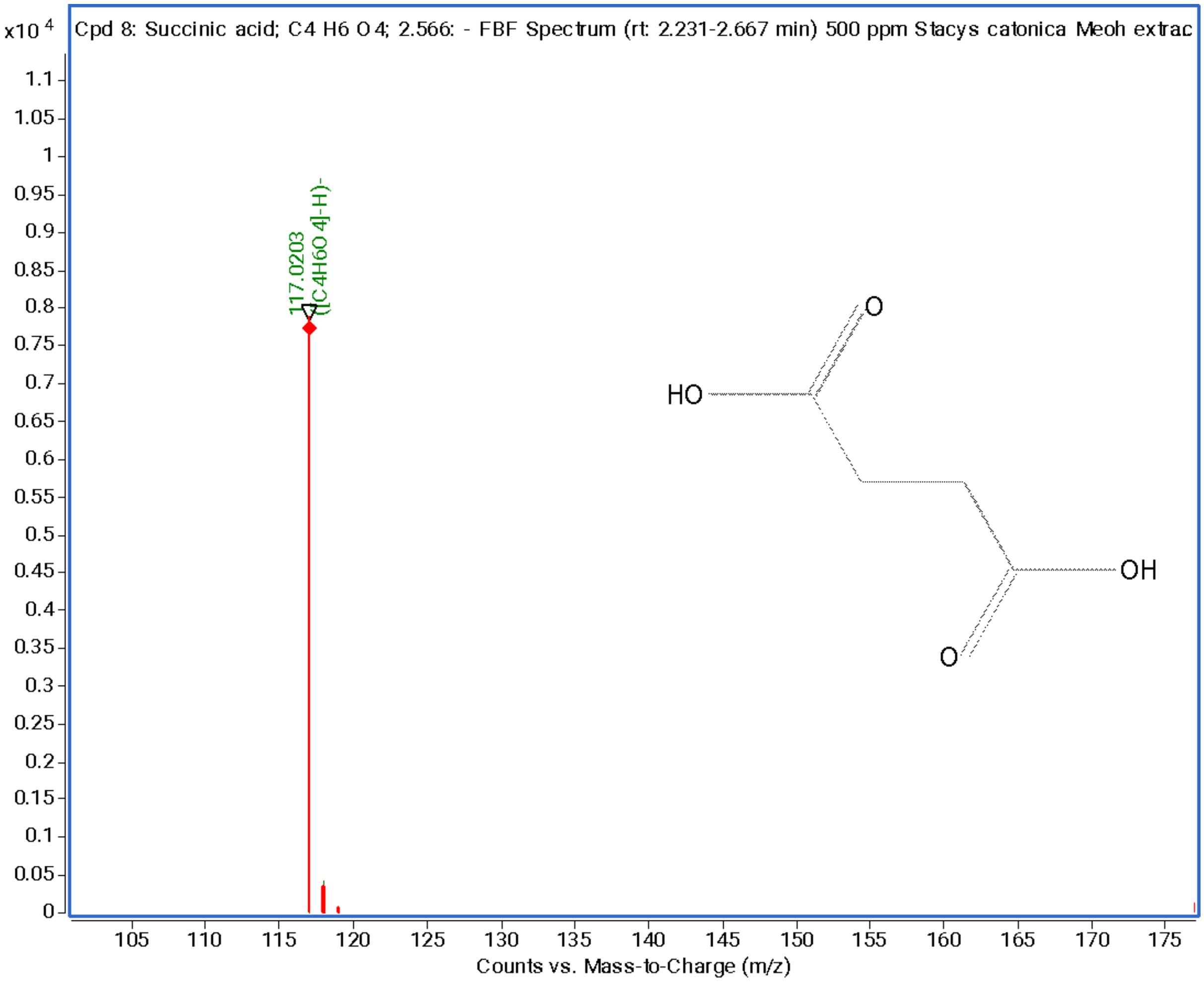

Figure S1. Negative ESI-MS full-scan spectrum of succinic acid.

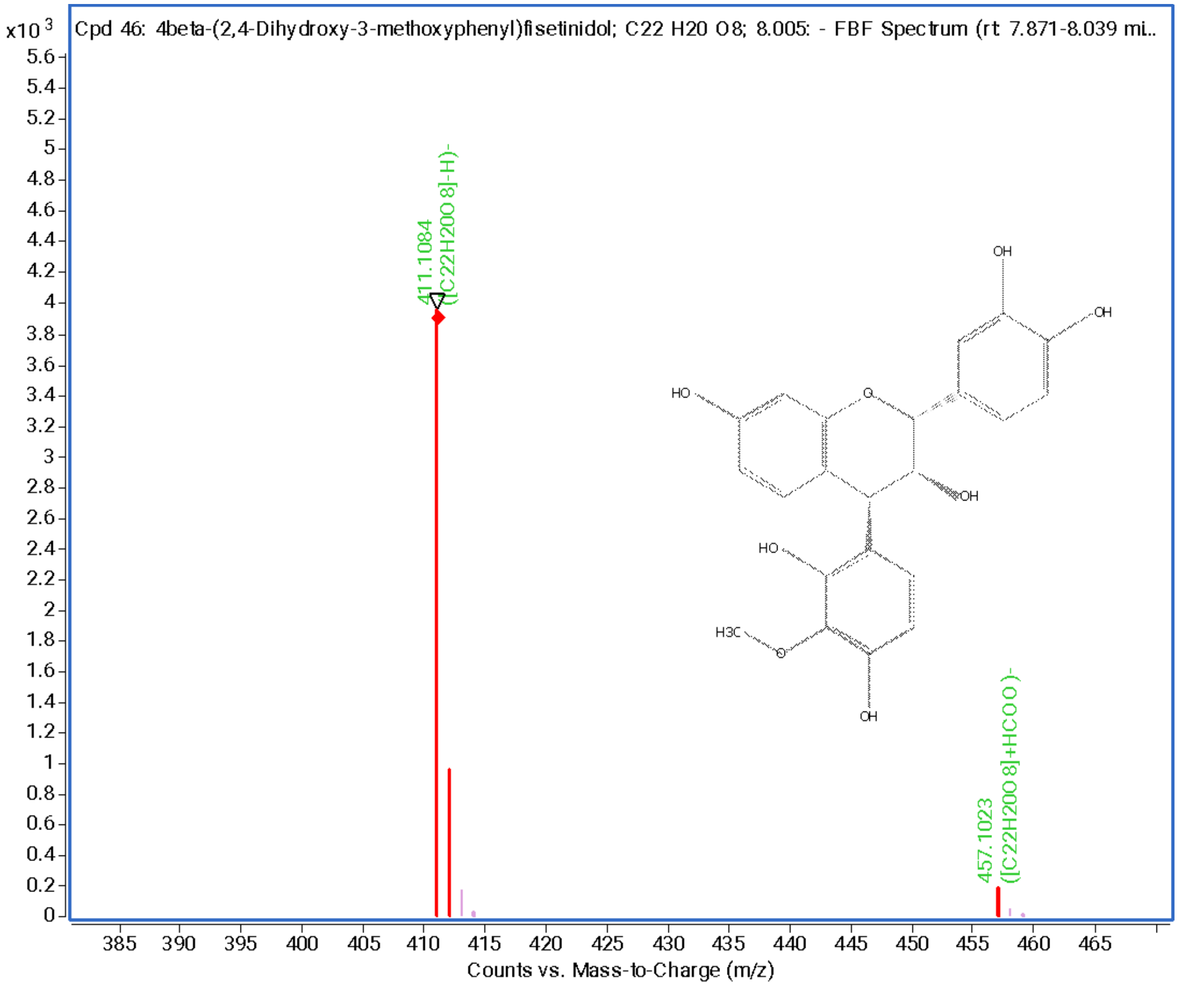

Figure S2. Negative ESI-MS full-scan spectrum of 4β-(2,4-dihydroxy-3-methoxyphenyl)fisetinidol.

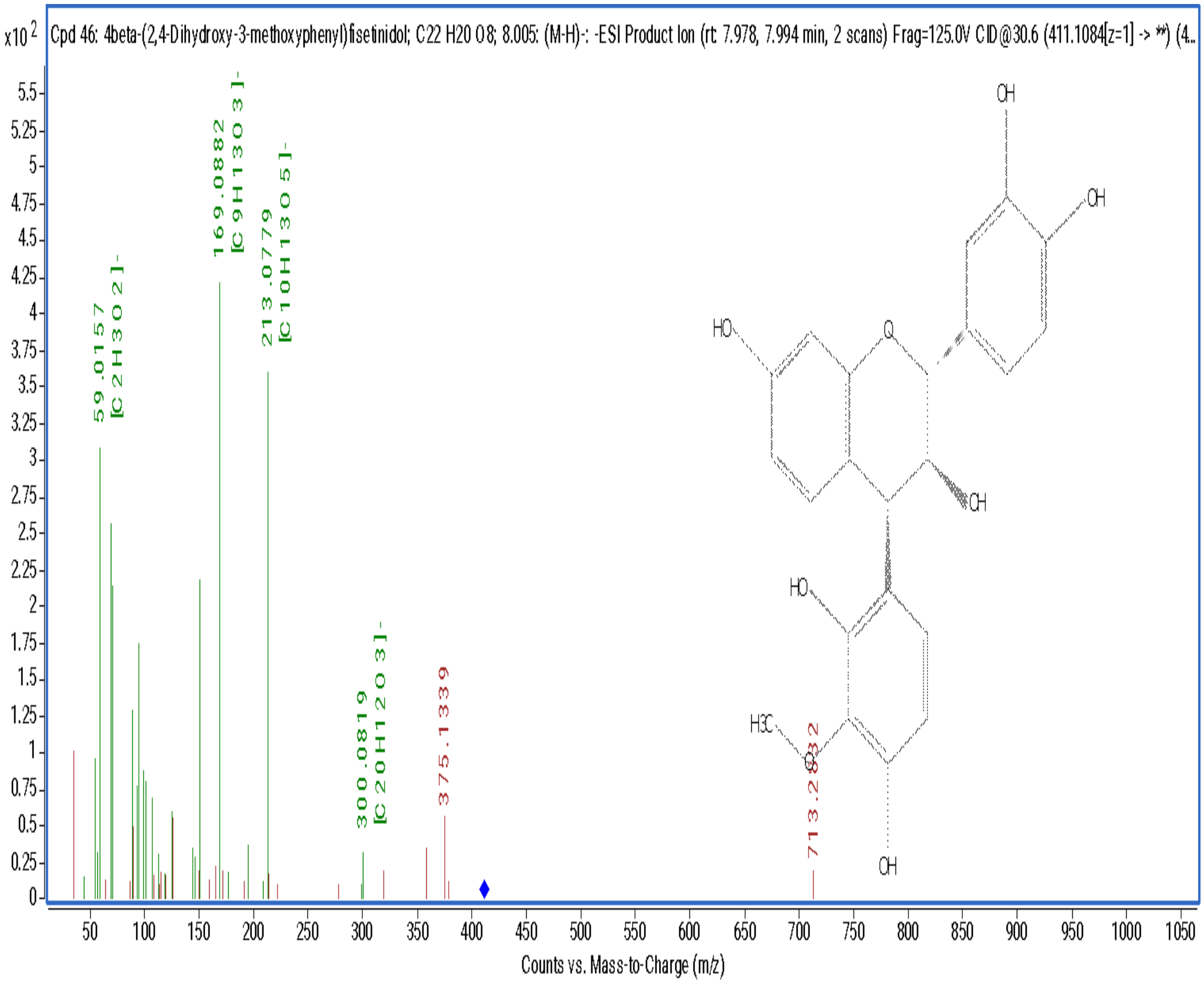

Figure S3. Negative-ion ESI-MS/MS product-ion spectrum of 4β-(2,4-dihydroxy-3-methoxyphenyl)fisetinidol.

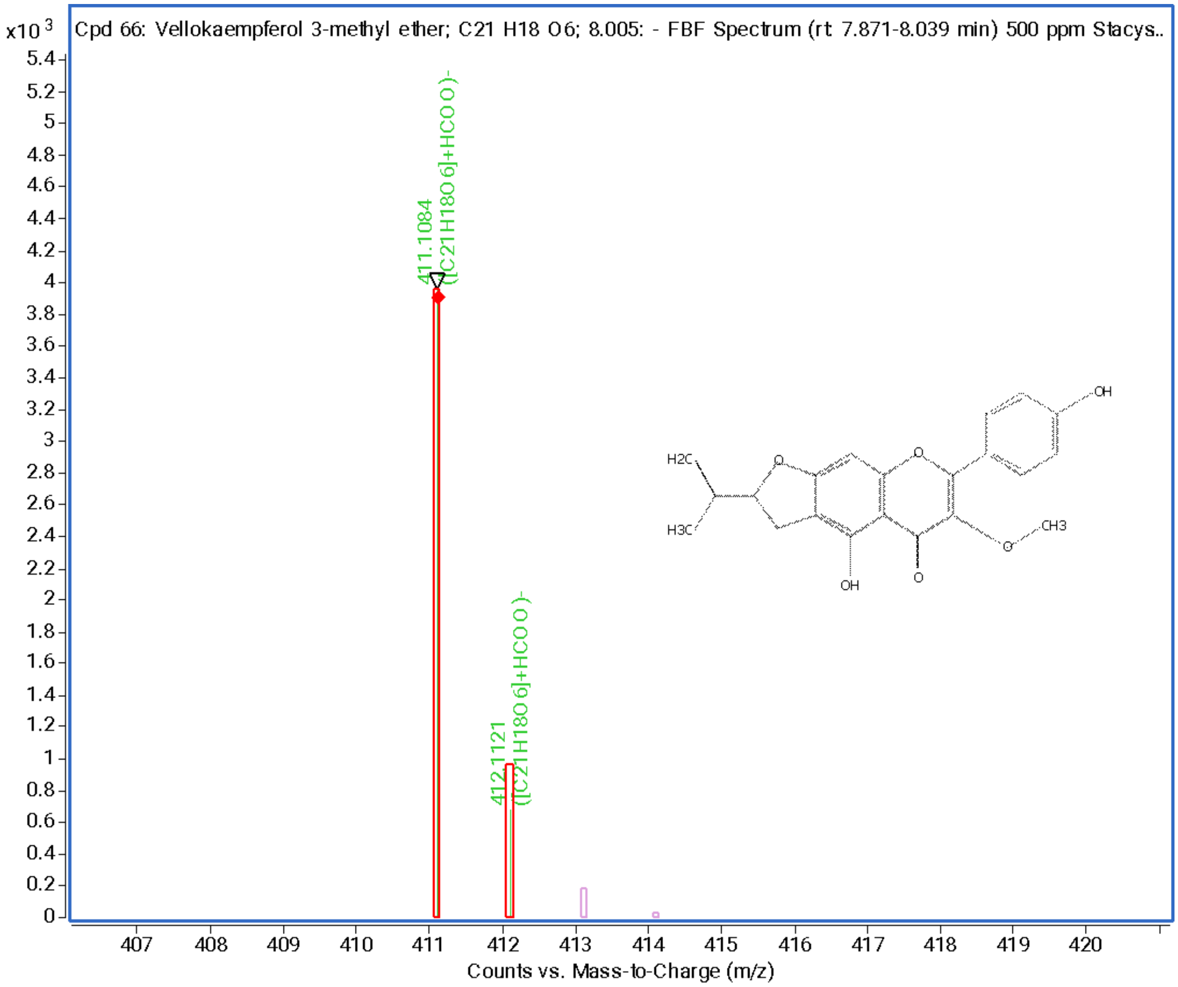

Figure S4. Negative ESI-MS full-scan spectrum of vellokaempferol 3-methyl ether

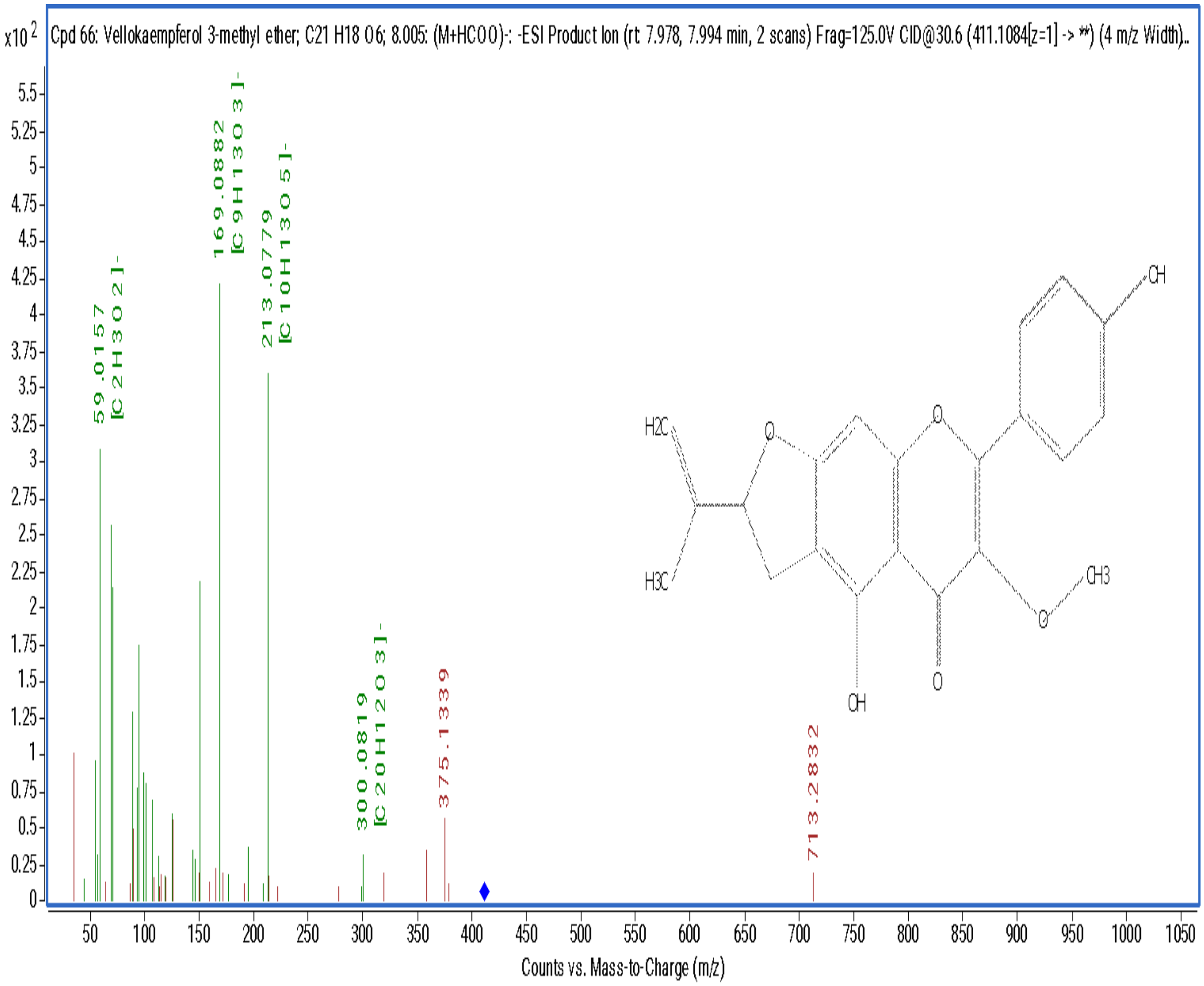

Figure S5. Negative-ion ESI-MS/MS product-ion spectrum of vellokaempferol 3-methyl ether

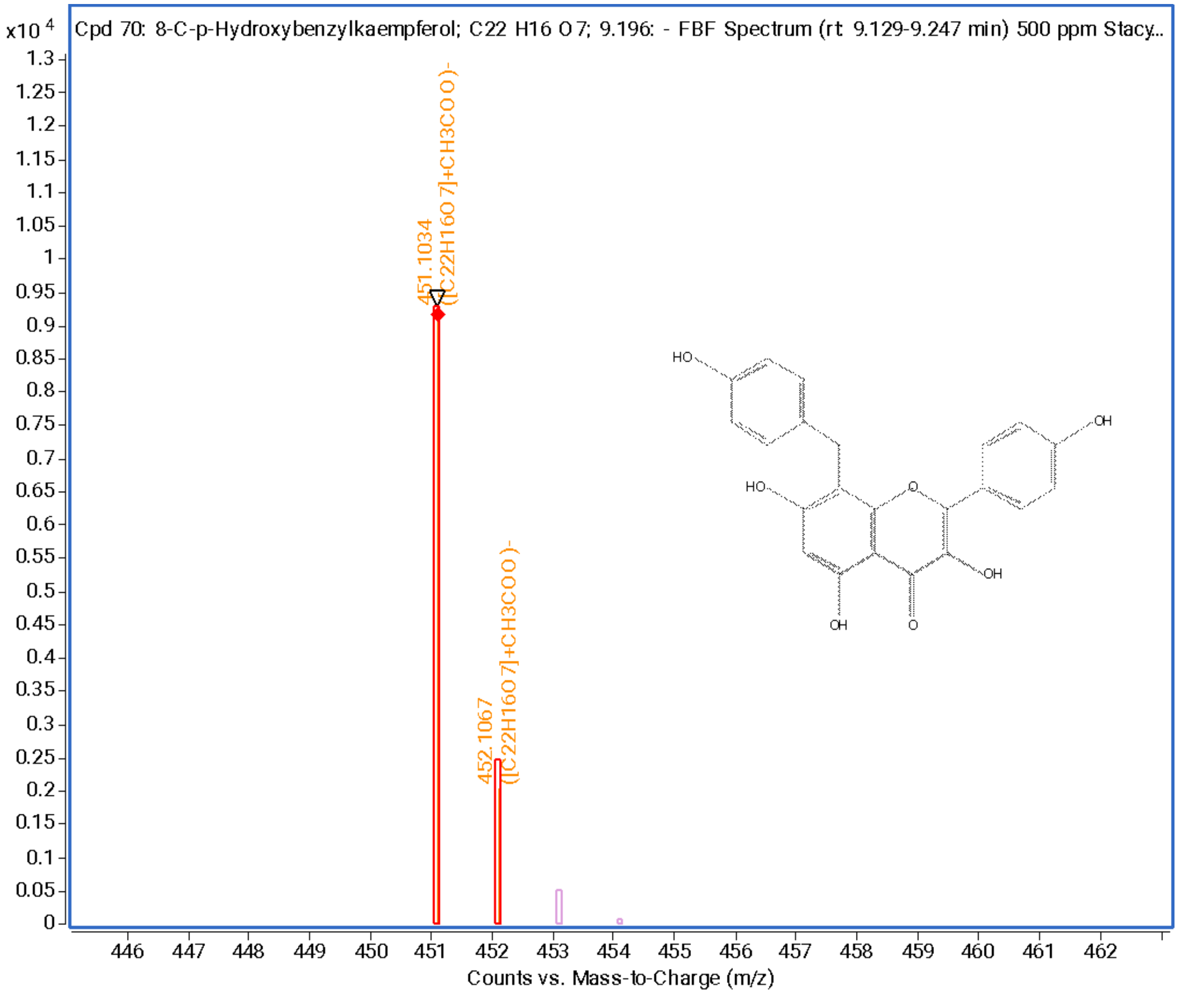

Figure S6. Negative ESI-MS full-scan spectrum of 8-C-p-hydroxybenzylkaempferol

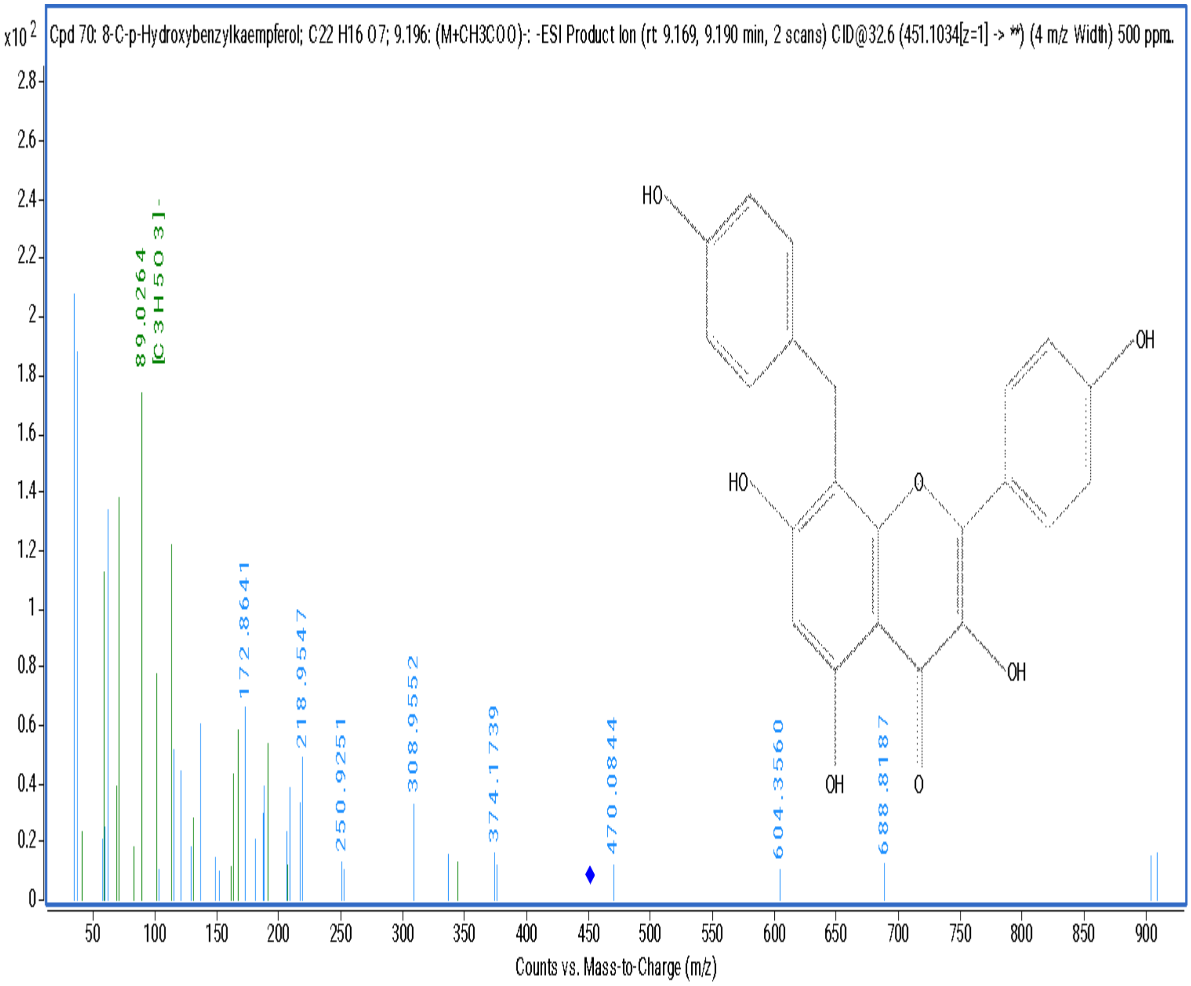

Figure S7. Negative-ion ESI-MS/MS product-ion spectrum of 8-C-p-hydroxybenzylkaempferol

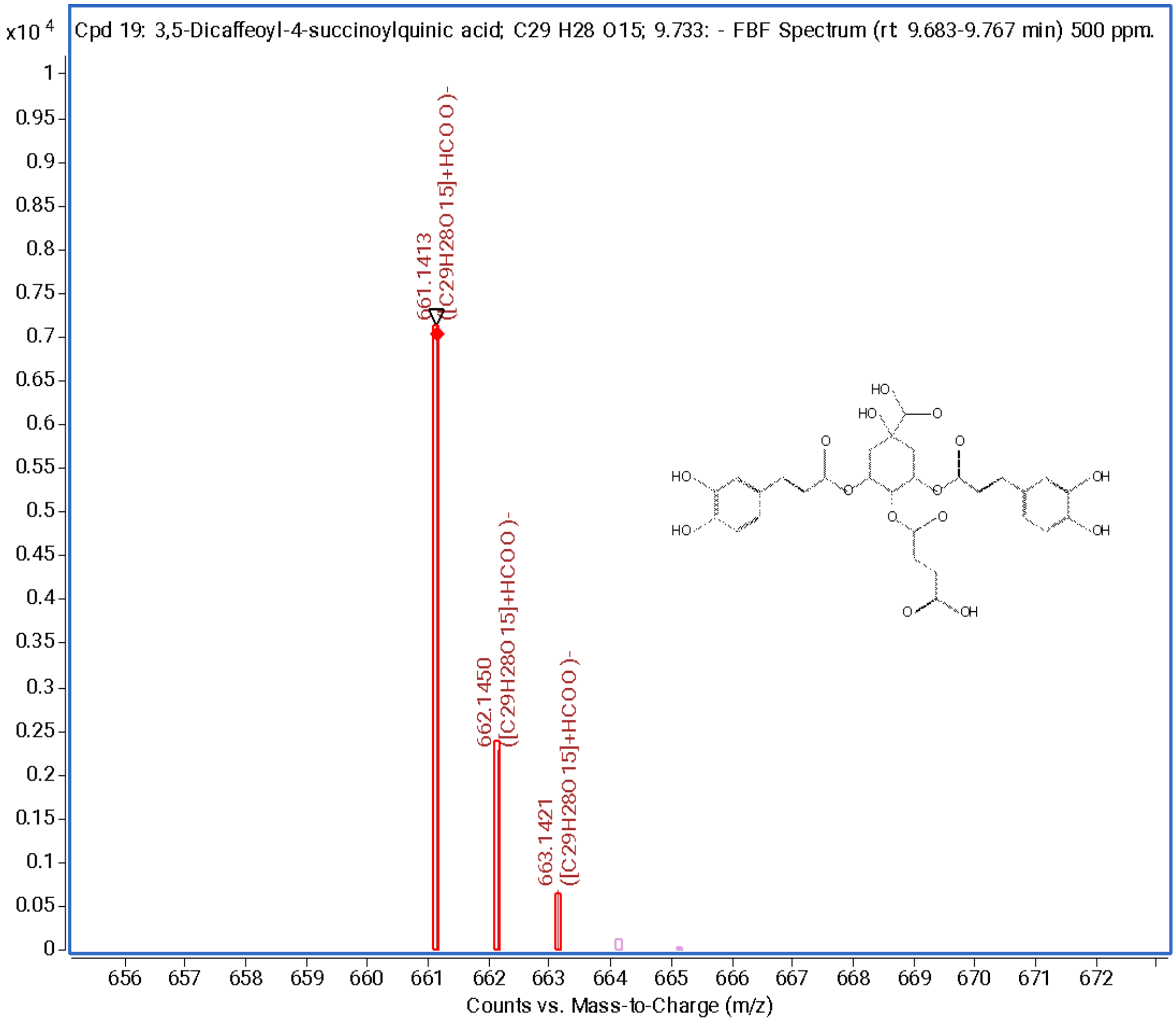

Figure S8. Negative ESI-MS full-scan spectrum of 3,5-dicaffeoyl-4-succinoylquinic acid

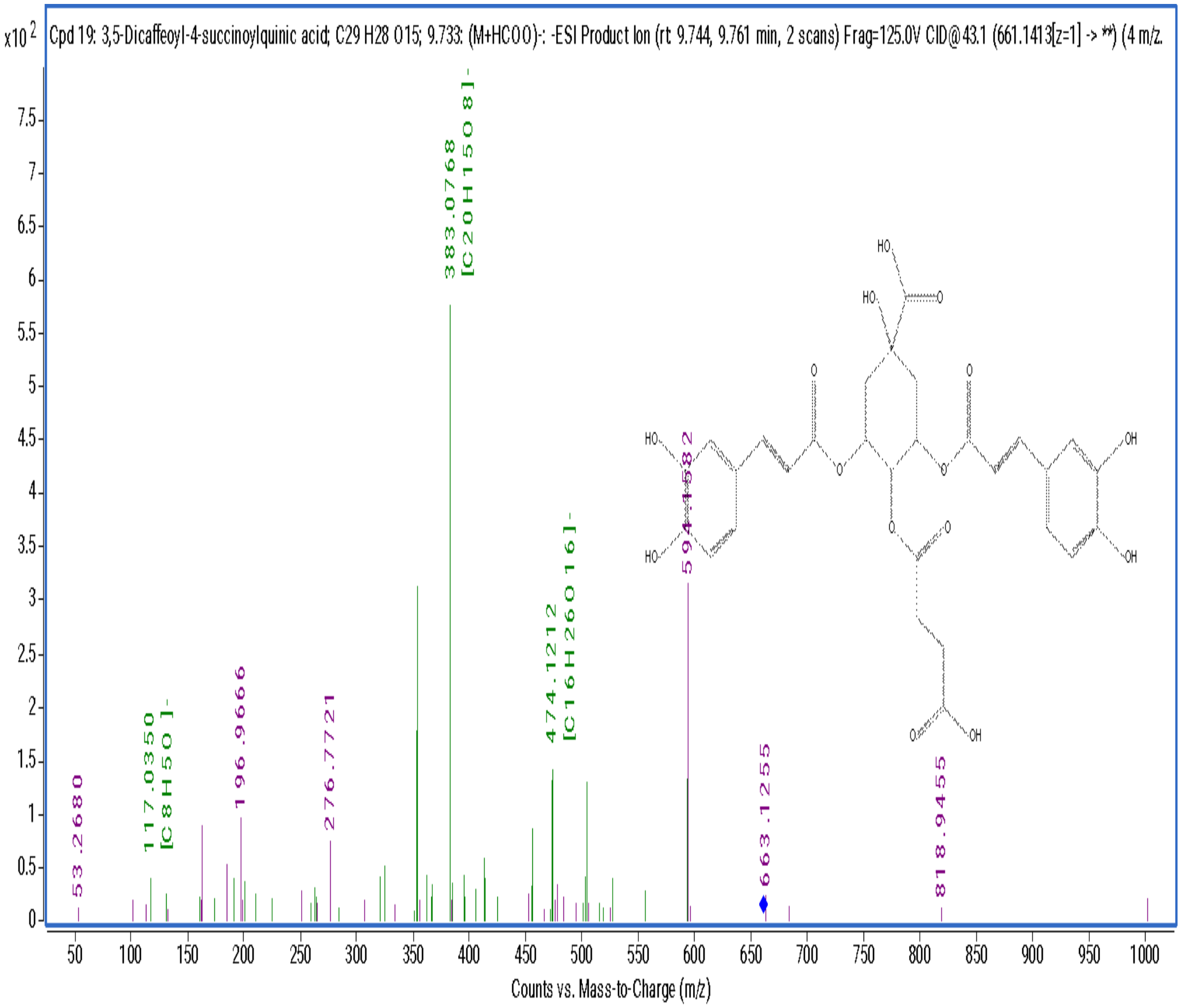

Figure S9. Negative-ion ESI-MS/MS product-ion spectrum of 3,5-dicaffeoyl-4-succinoylquinic acid

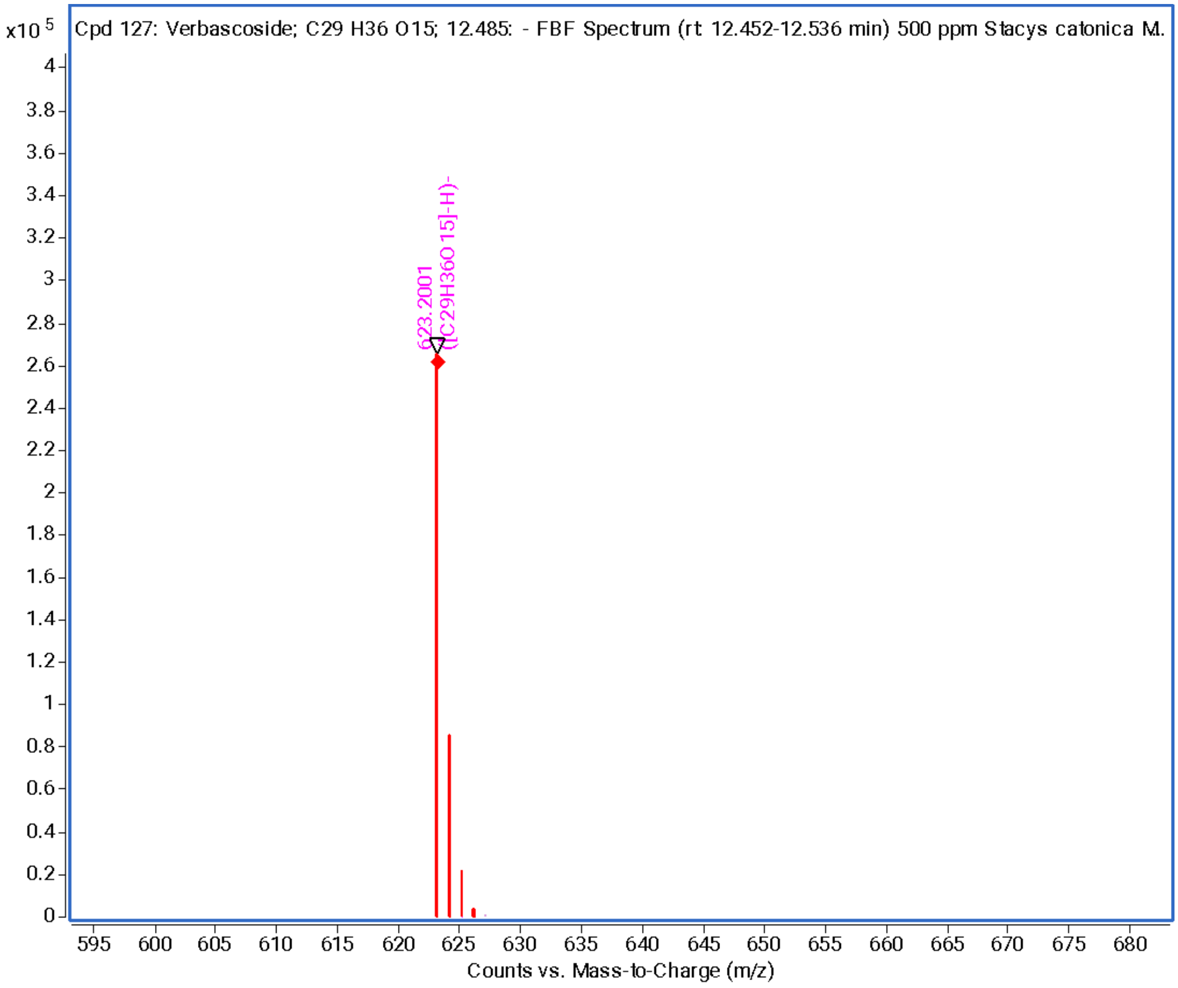

Figure S10. Negative ESI-MS full-scan spectrum of verbascoside

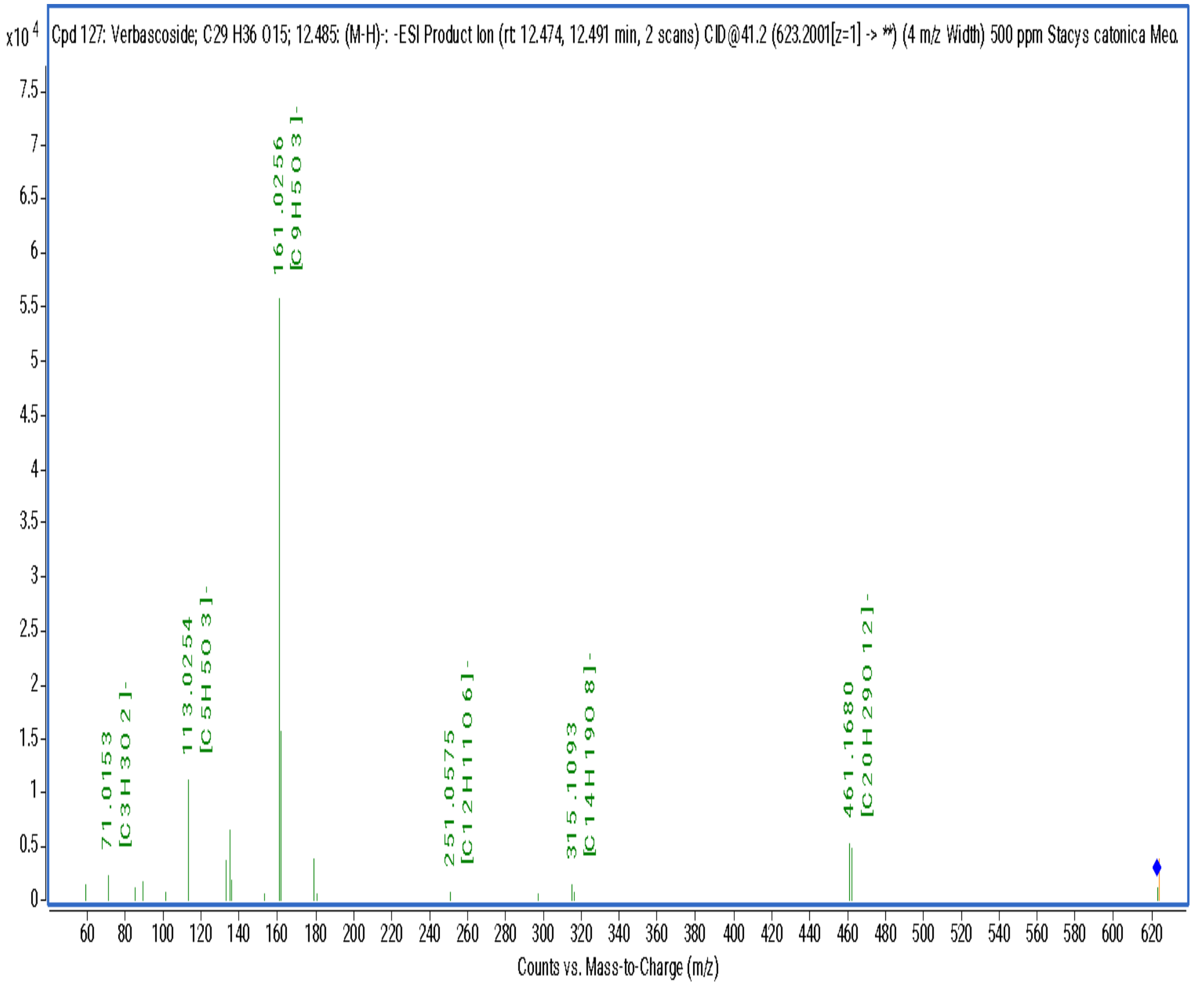

Figure S11. Negative-ion ESI-MS/MS product-ion spectrum of verbascoside

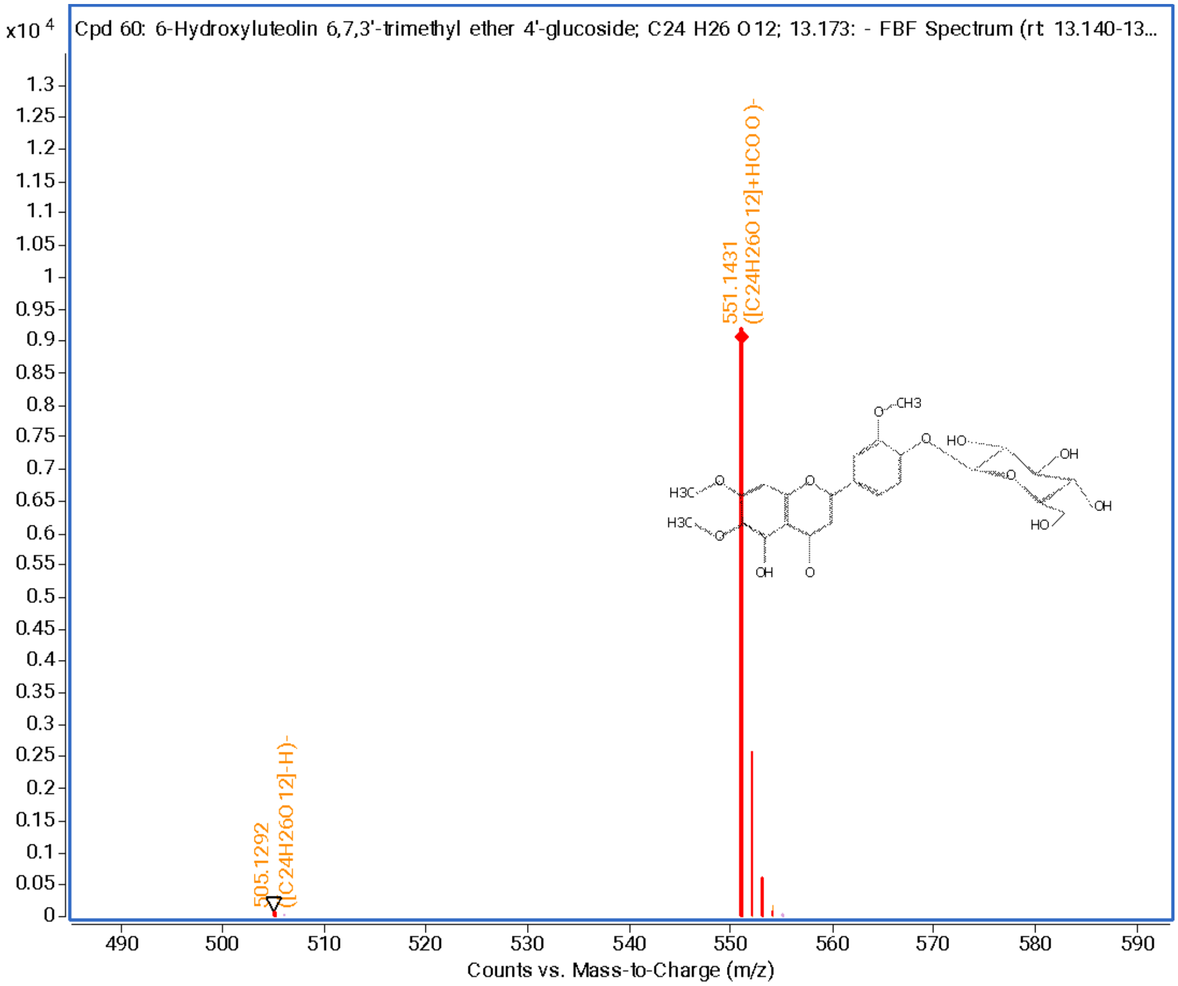

Figure S12. Negative ESI-MS full-scan spectrum of 6-hydroxyluteolin 6,7,3′-trimethyl ether 4′-glucoside

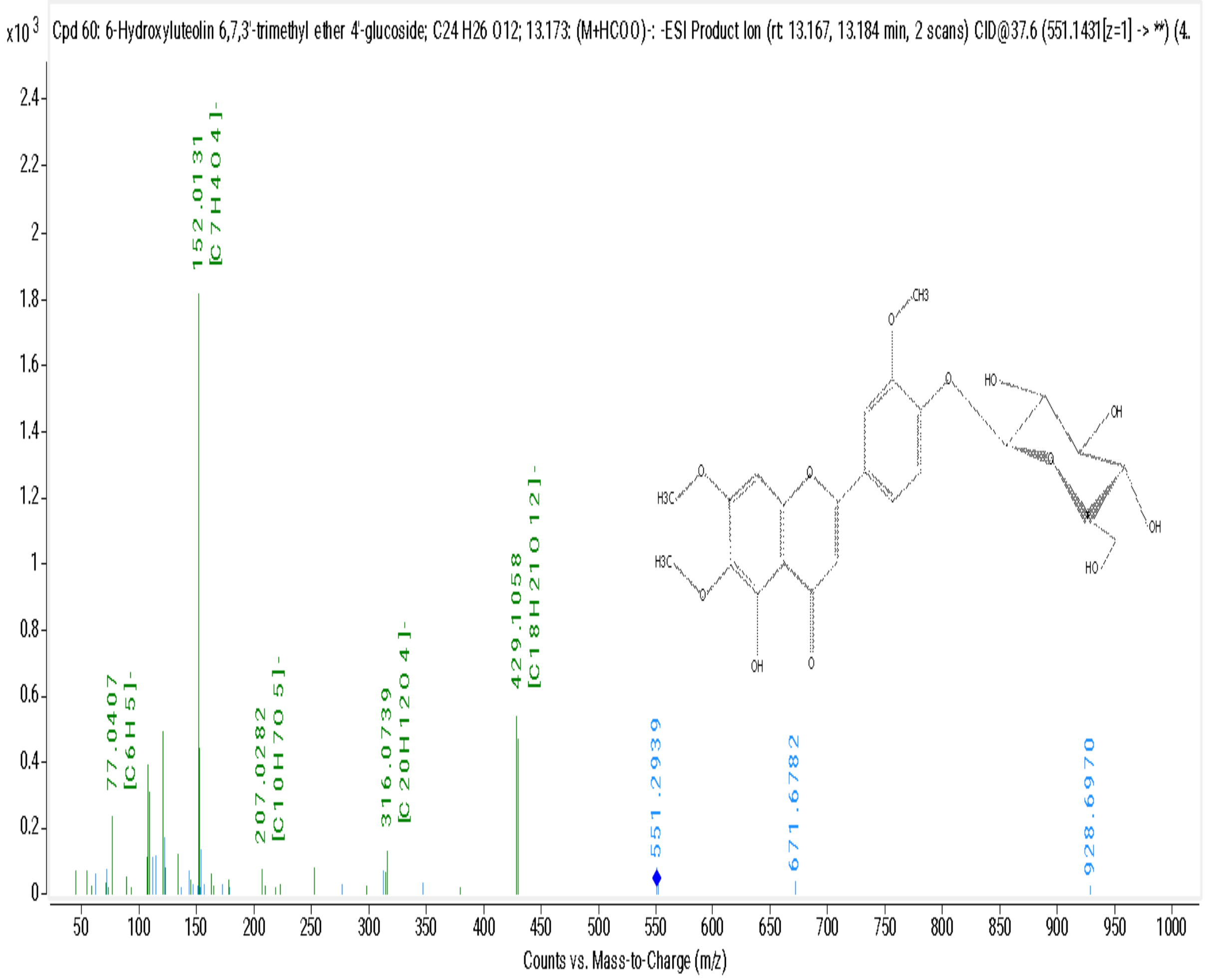

Figure S13. Negative-ion ESI-MS/MS product-ion spectrum of 6-hydroxyluteolin 6,7,3′-trimethyl ether 4′-glucoside

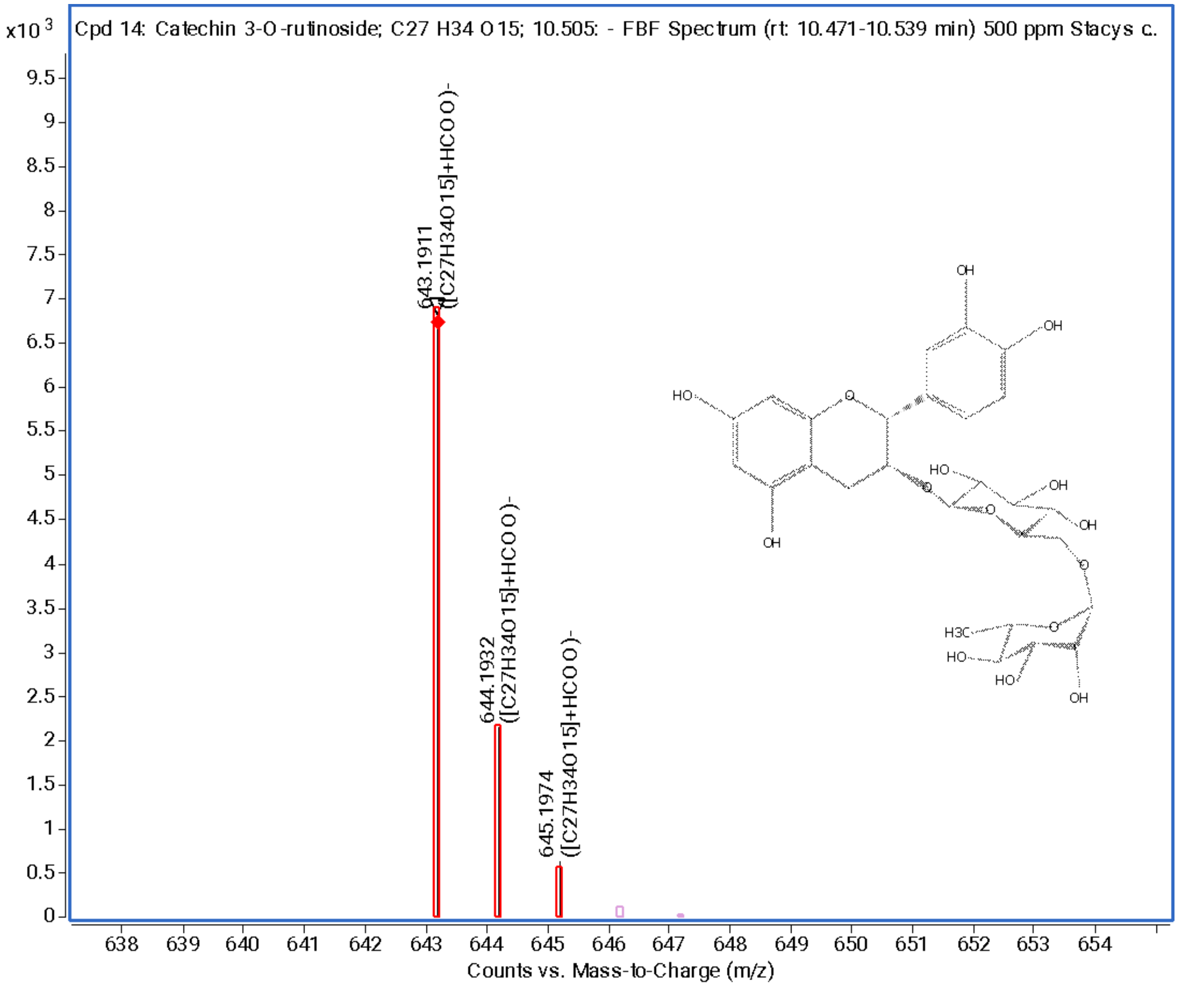

Figure S14. Negative ESI-MS full-scan spectrum of catechin 3-O-rutinoside

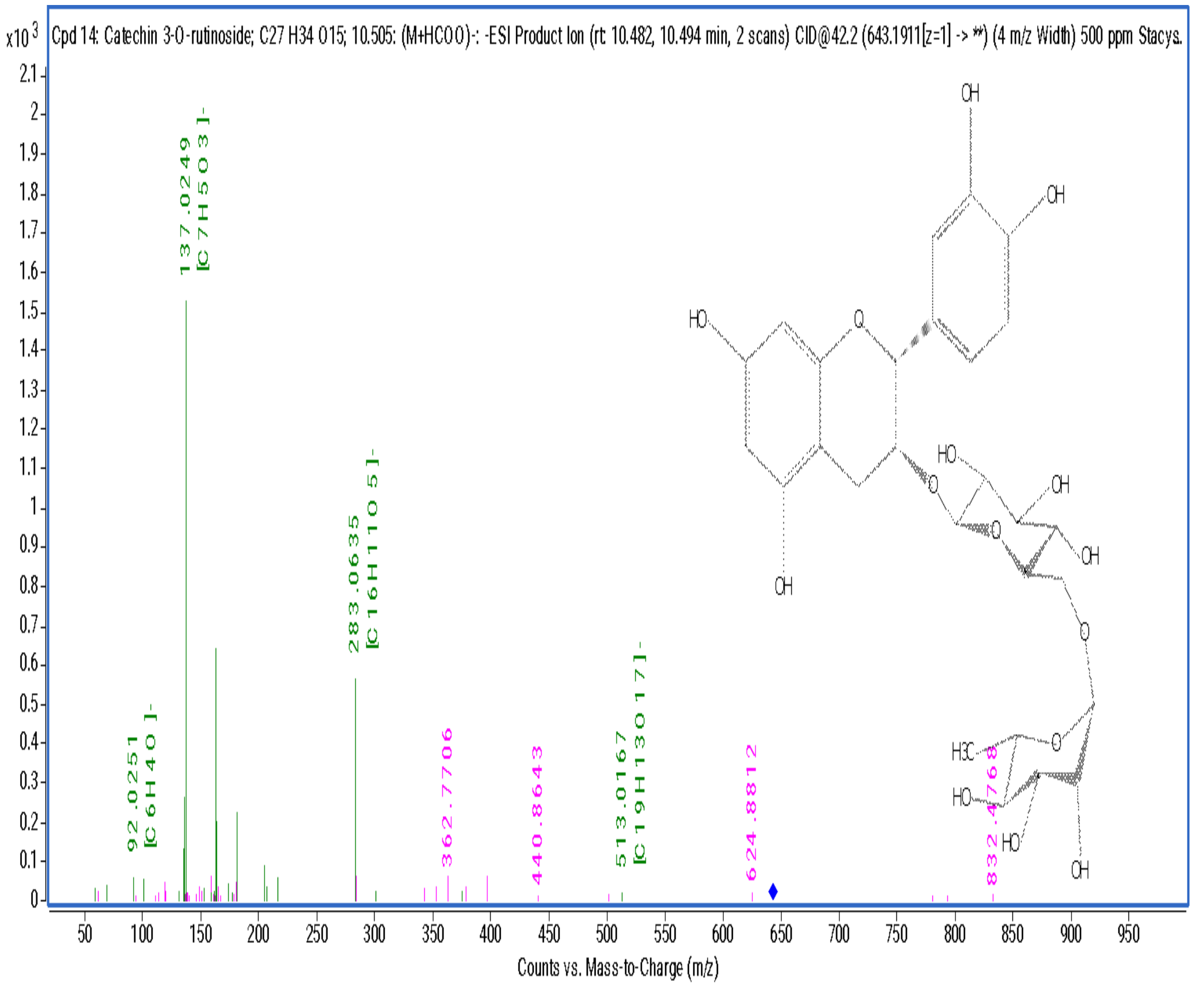

Figure S15. Negative-ion ESI-MS/MS product-ion spectrum of catechin 3-O-rutinoside

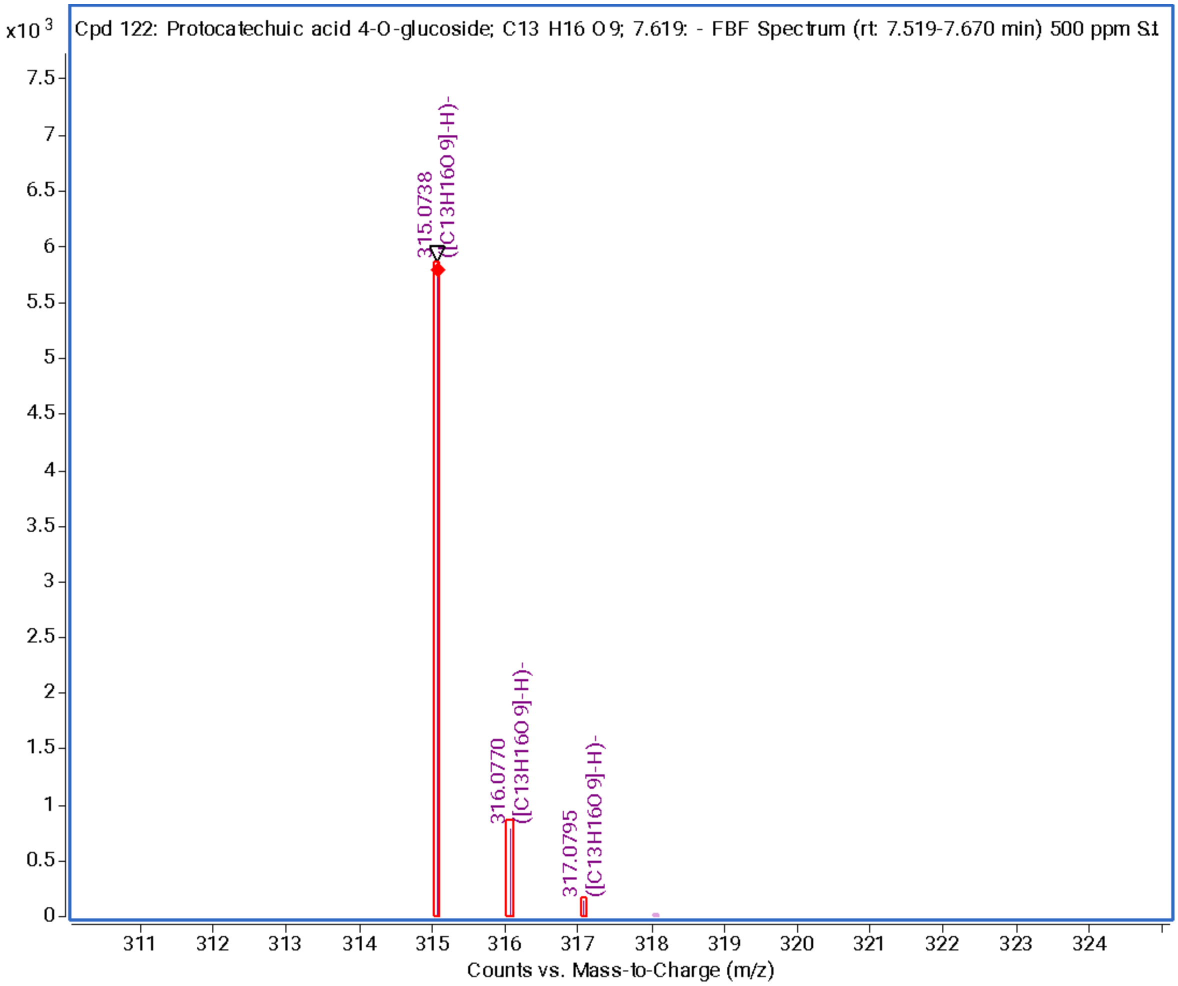

Figure S16. Negative ESI-MS full-scan spectrum of protocatechuic acid 4-O-glucoside

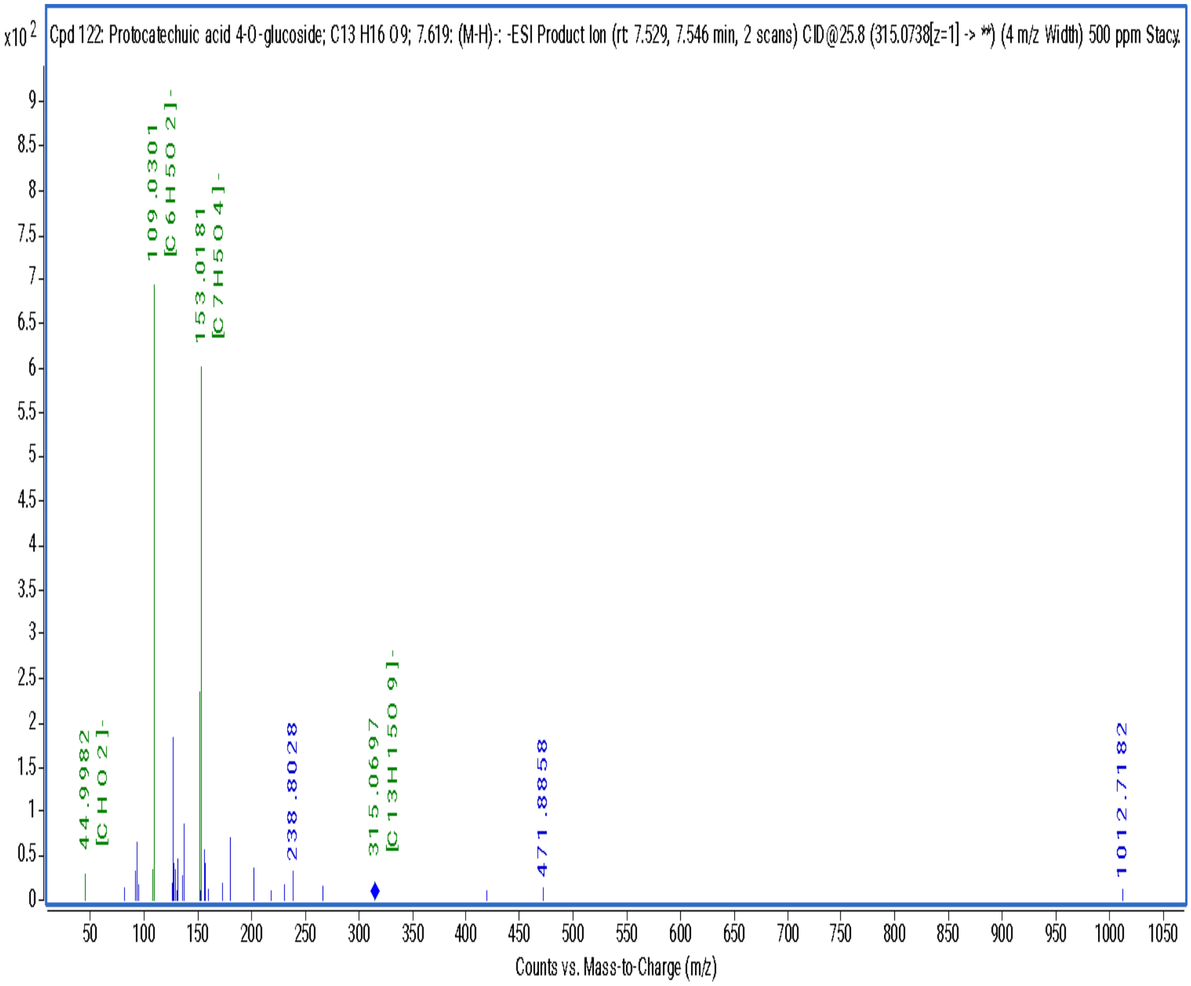

Figure S17. Negative-ion ESI-MS/MS product-ion spectrum of protocatechuic acid 4-O-glucoside

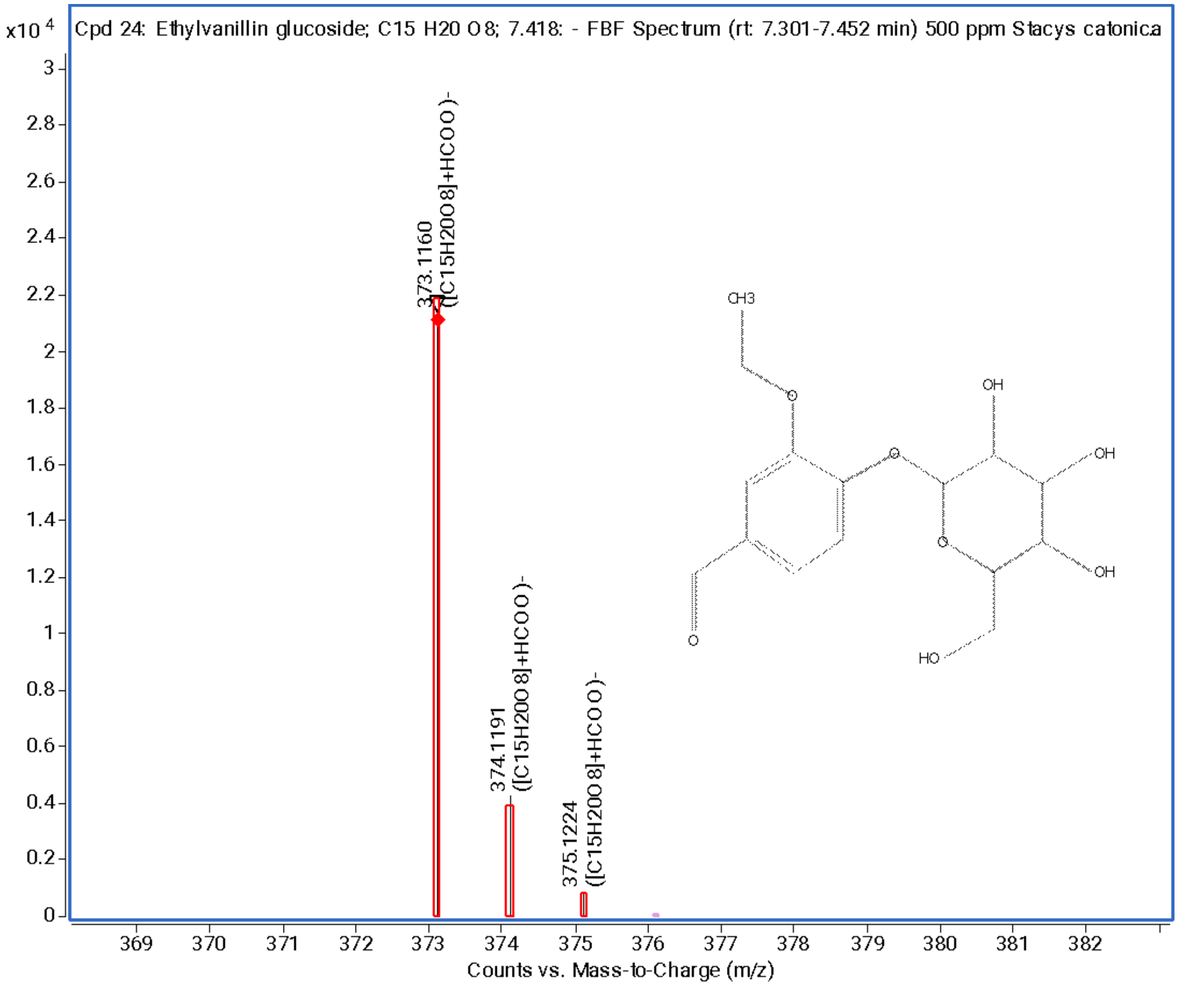

Figure S18. Negative ESI-MS full-scan spectrum of ethylvanillin glucoside

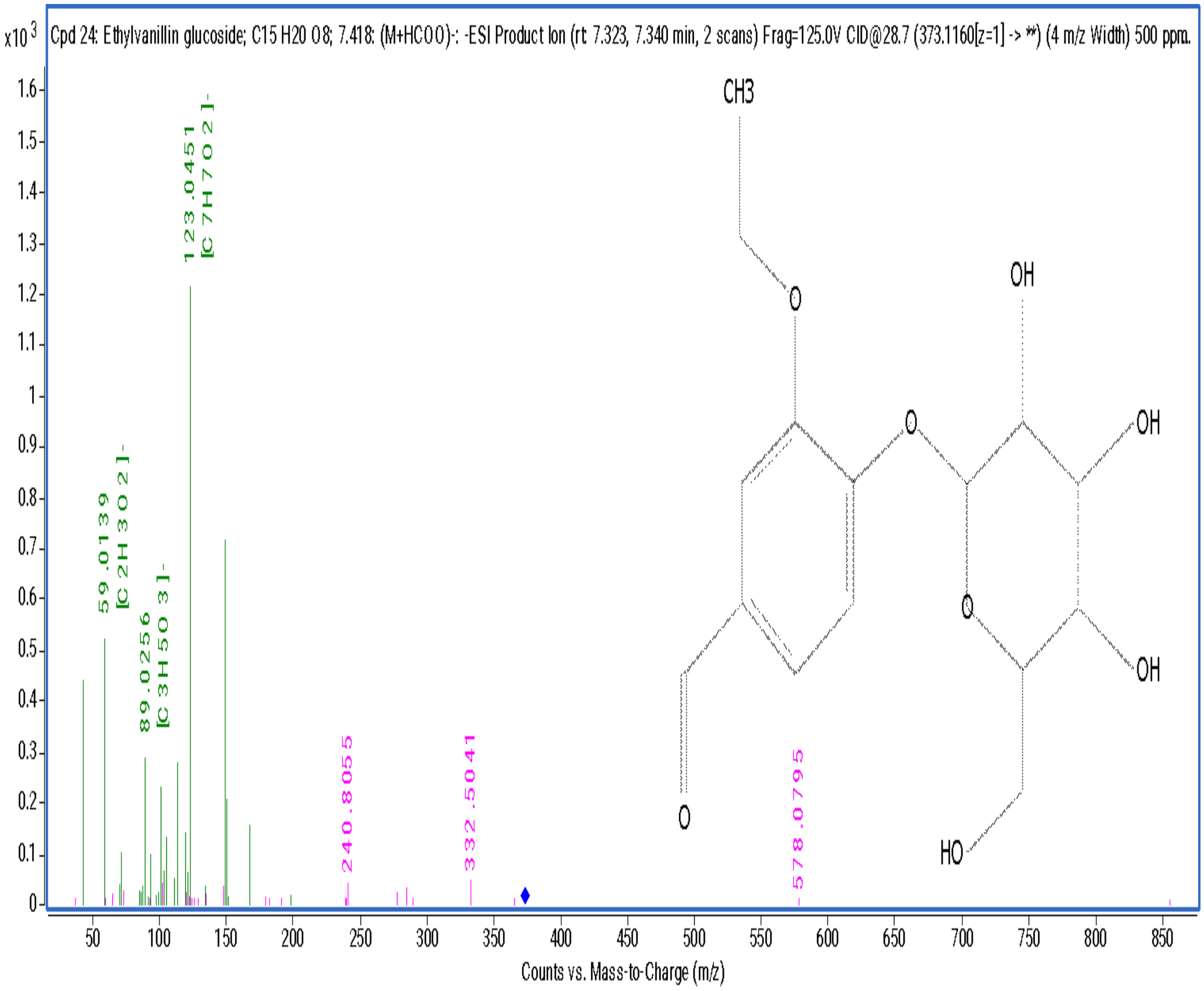

Figure S19. Negative-ion ESI-MS/MS product-ion spectrum of ethylvanillin glucoside

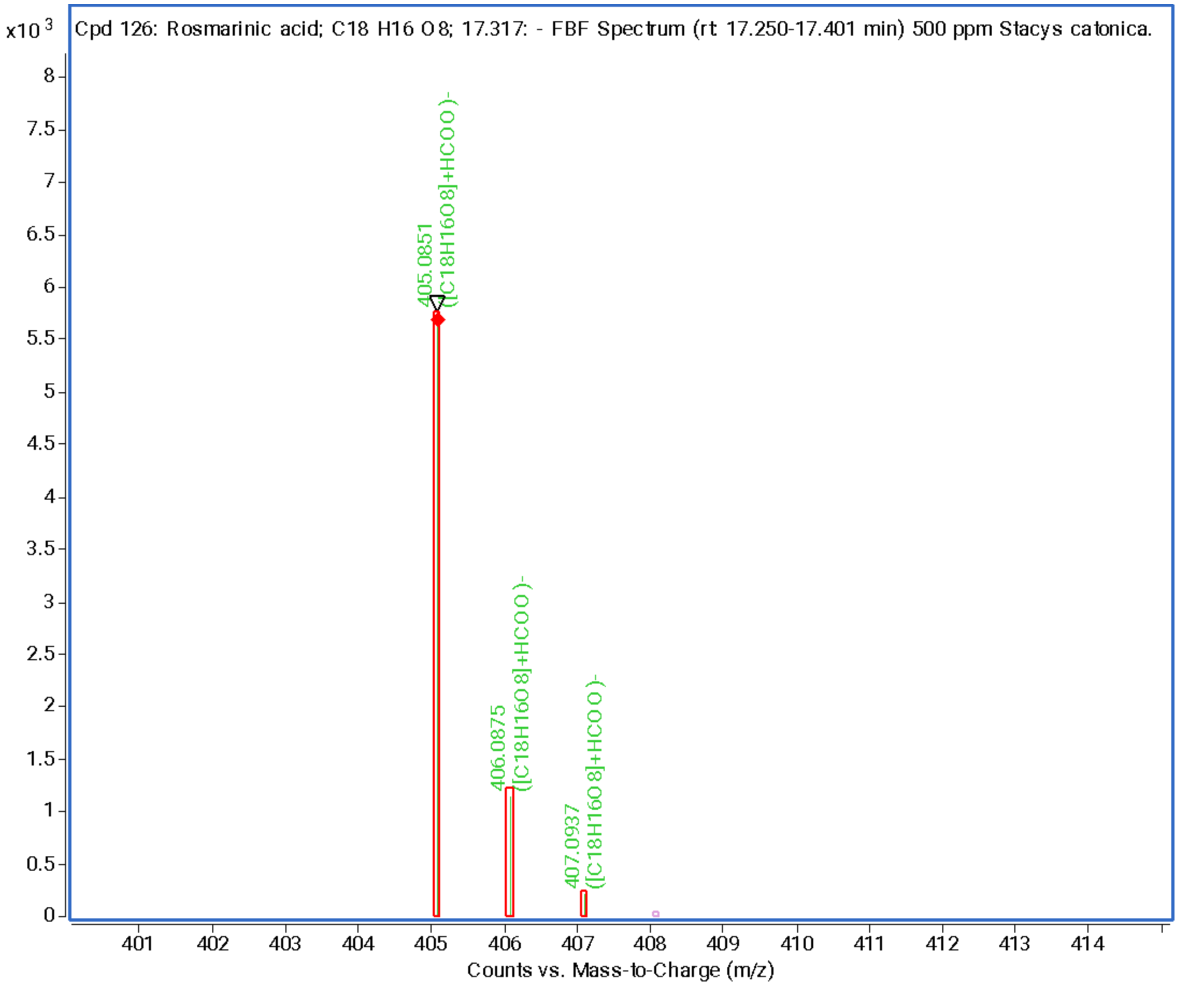

Figure S20. Negative ESI-MS full-scan spectrum of rosmarinic acid

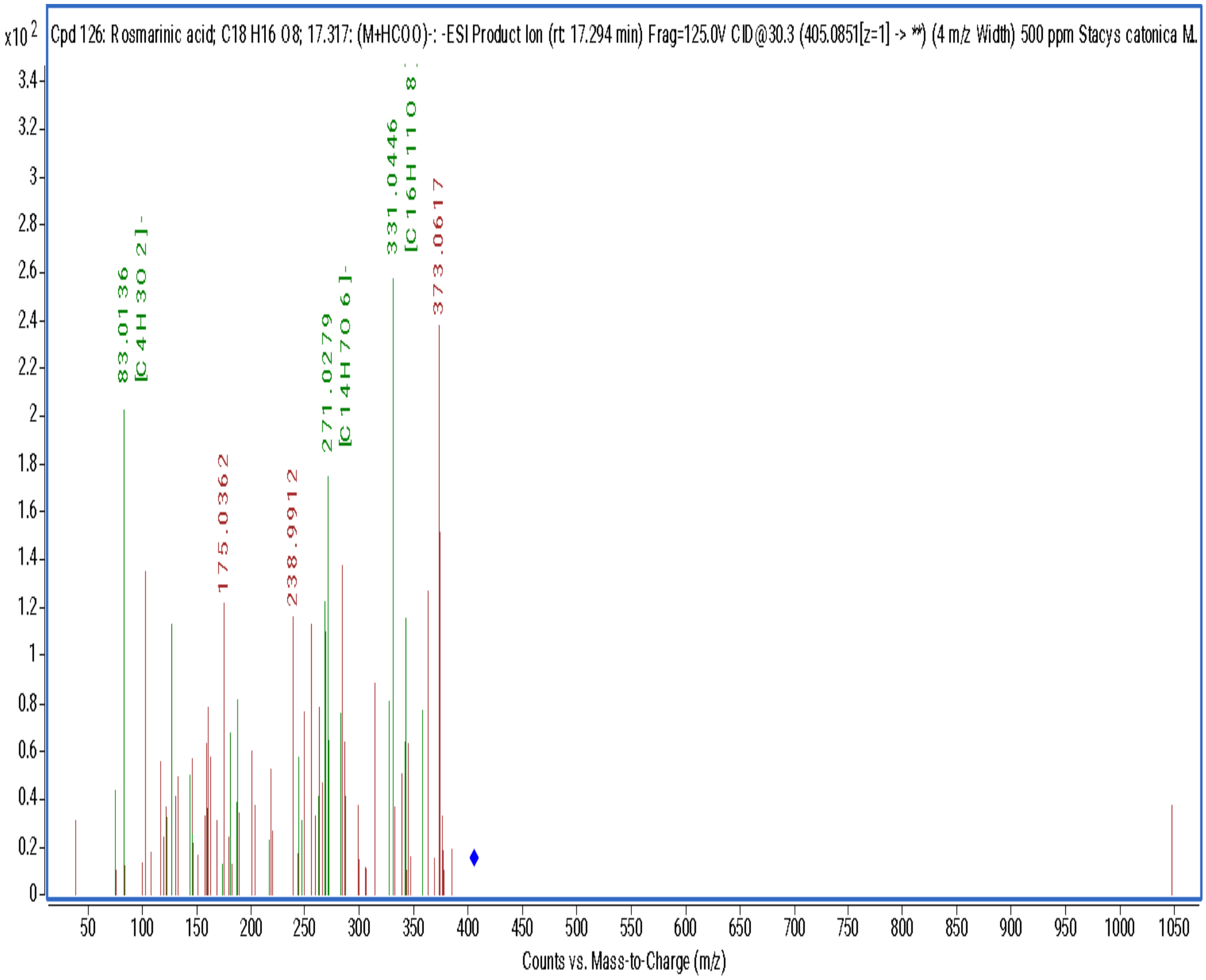

Figure S21. Negative-ion ESI-MS/MS product-ion spectrum of rosmarinic acid

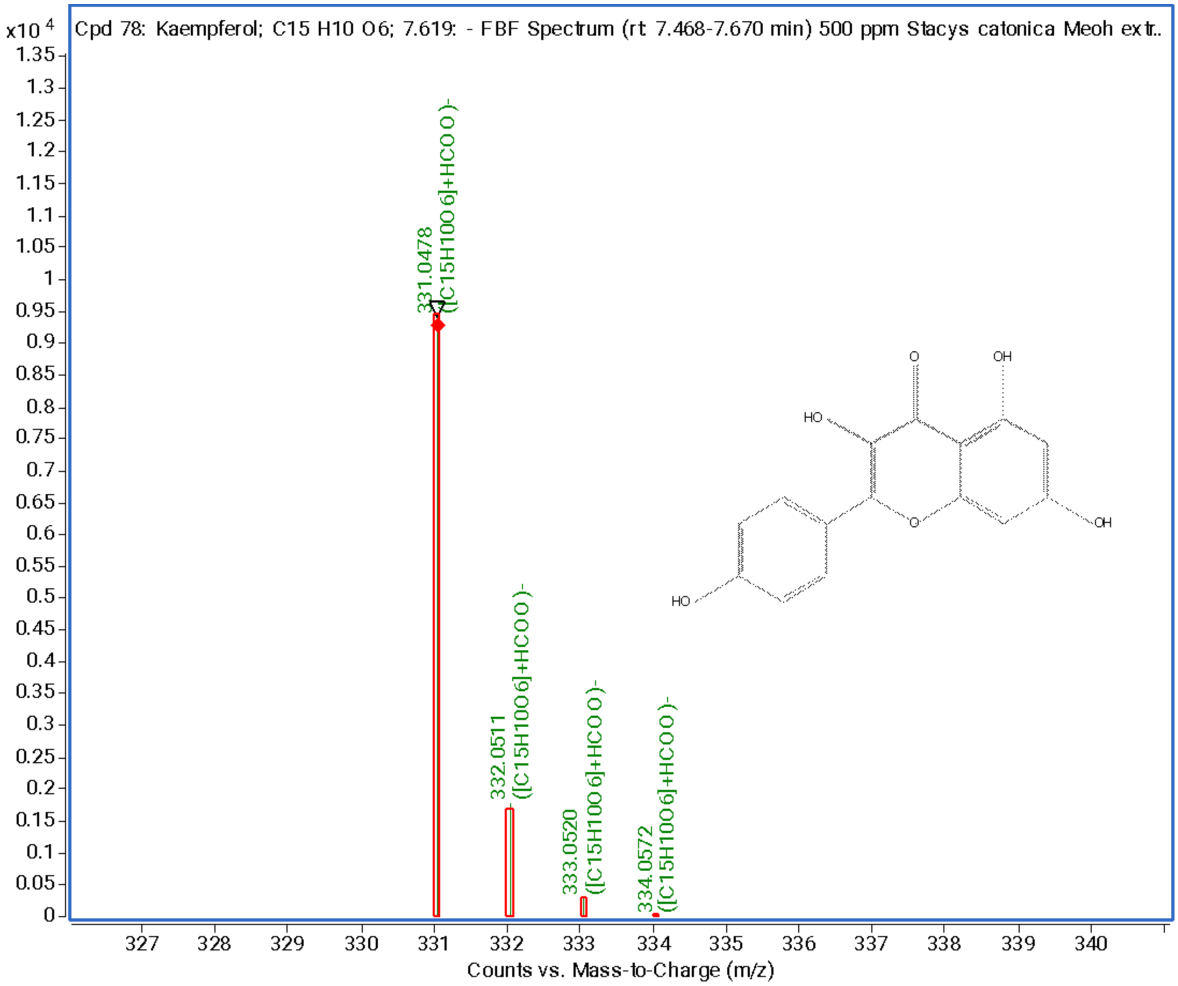

Figure S22. Negative ESI-MS full-scan spectrum of kaempferol

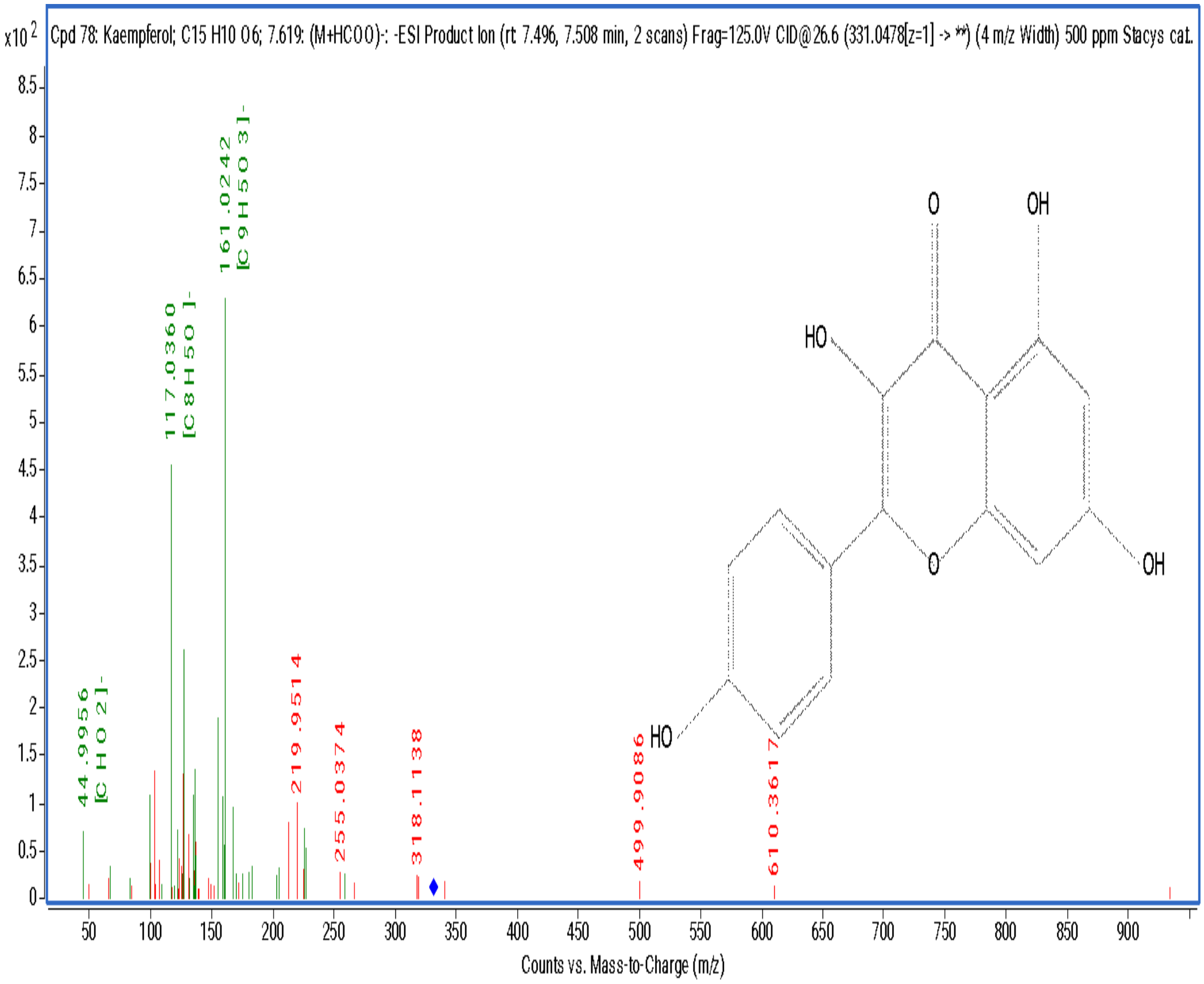

Figure S23. Negative-ion ESI-MS/MS product-ion spectrum of kaempferol

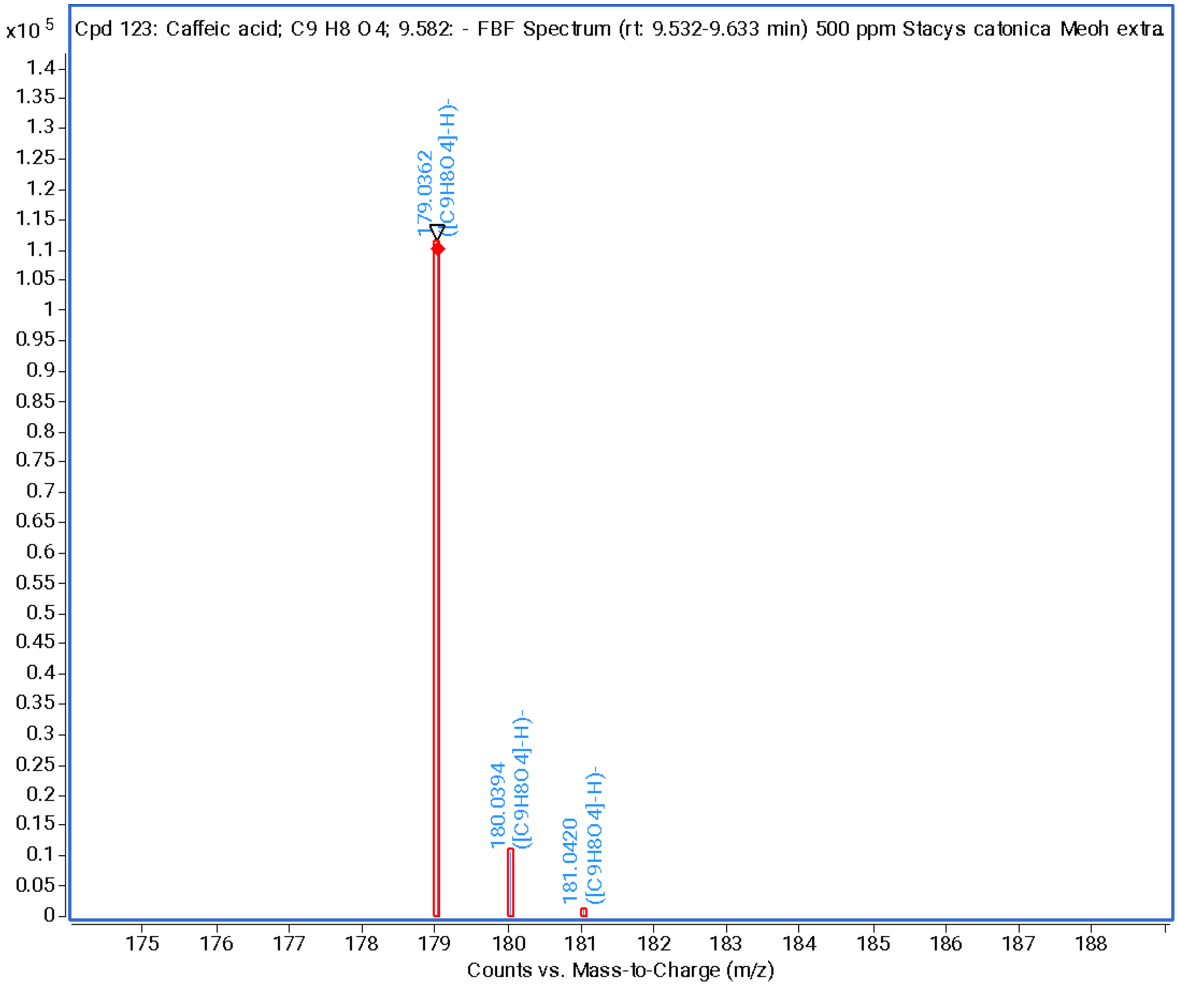

Figure S24. Negative ESI-MS full-scan spectrum of caffeic acid

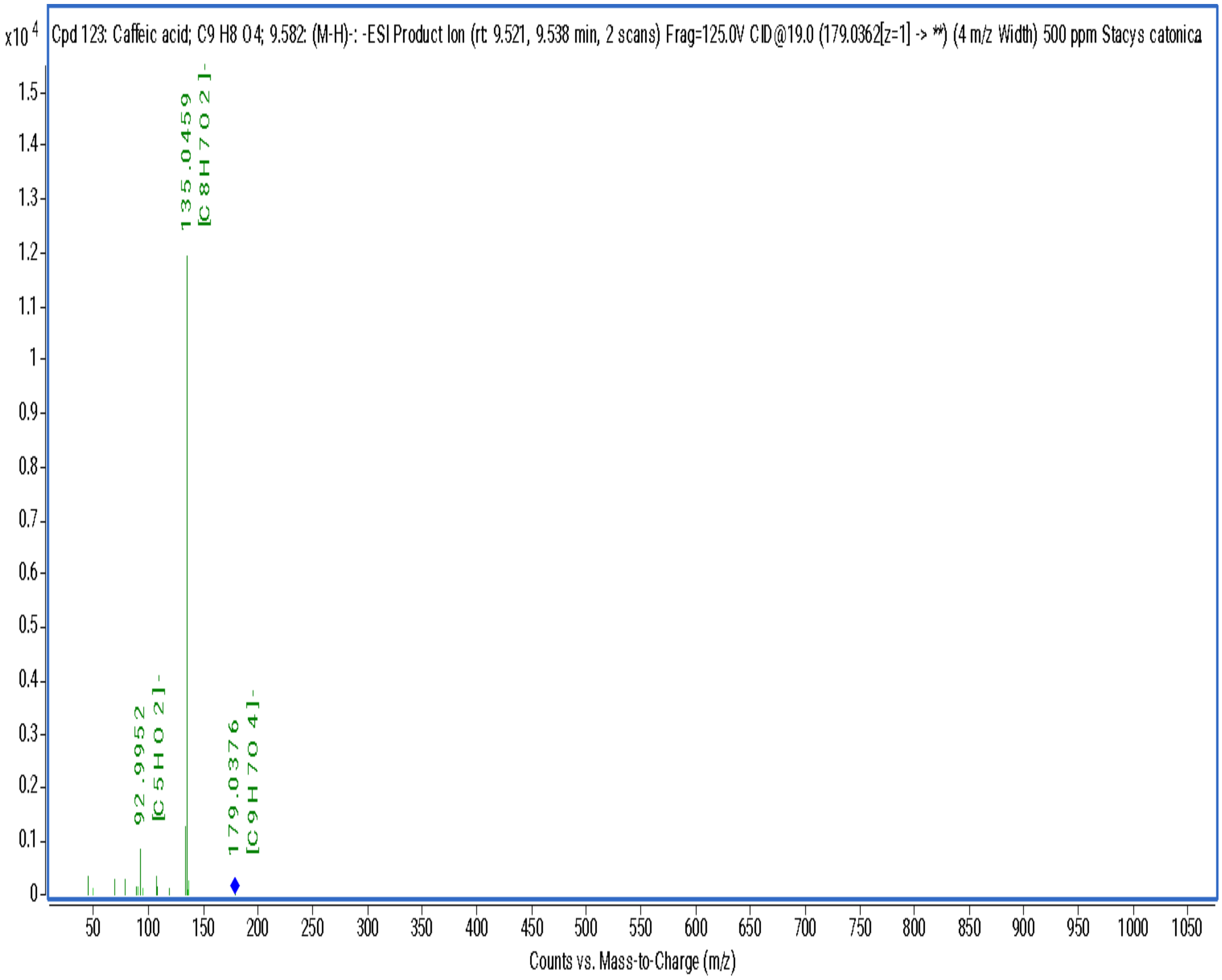

Figure S25. Negative-ion ESI-MS/MS product-ion spectrum of caffeic acid

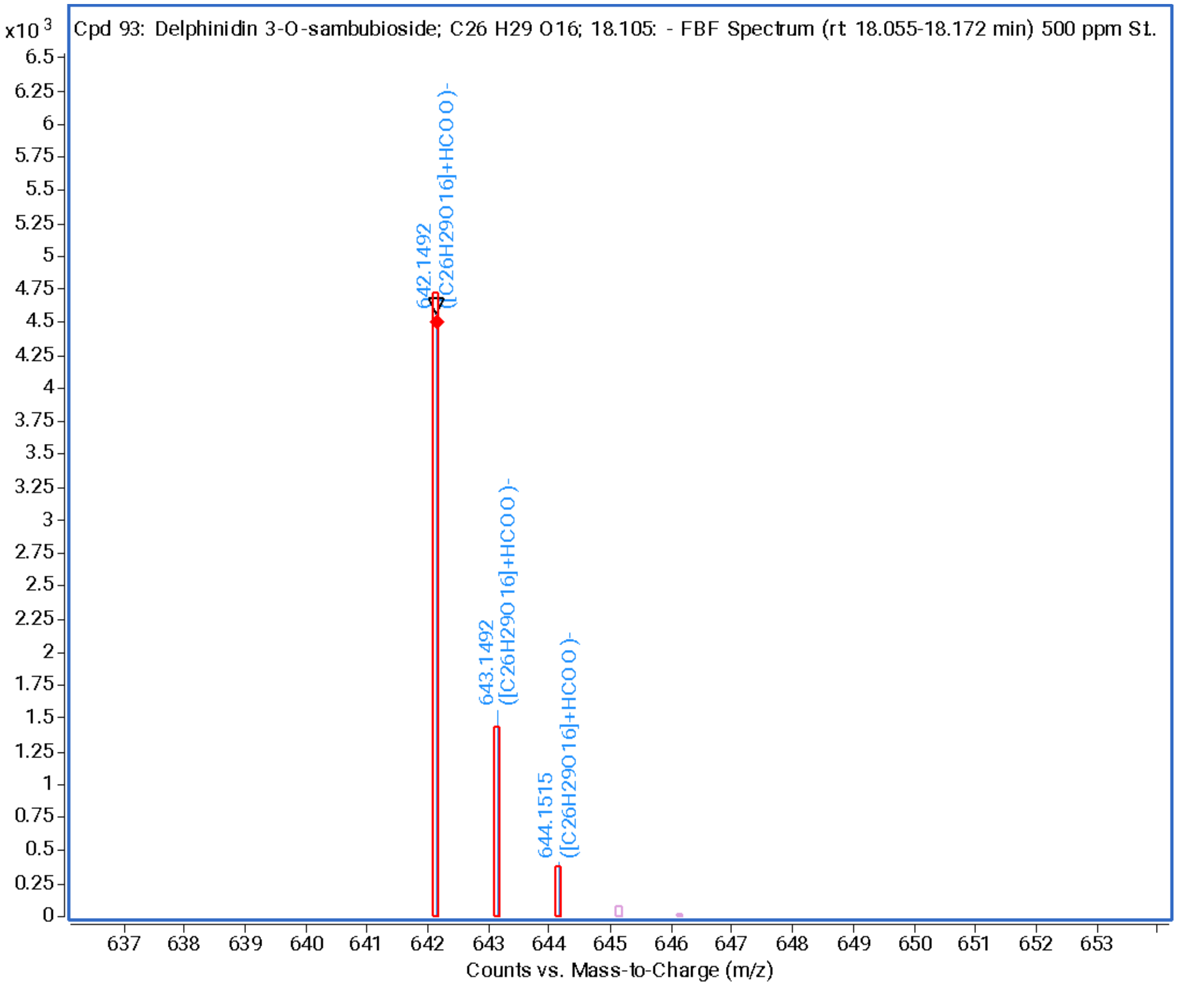

Figure S26. Negative ESI-MS full-scan spectrum of delphinidin 3-O-sambubioside

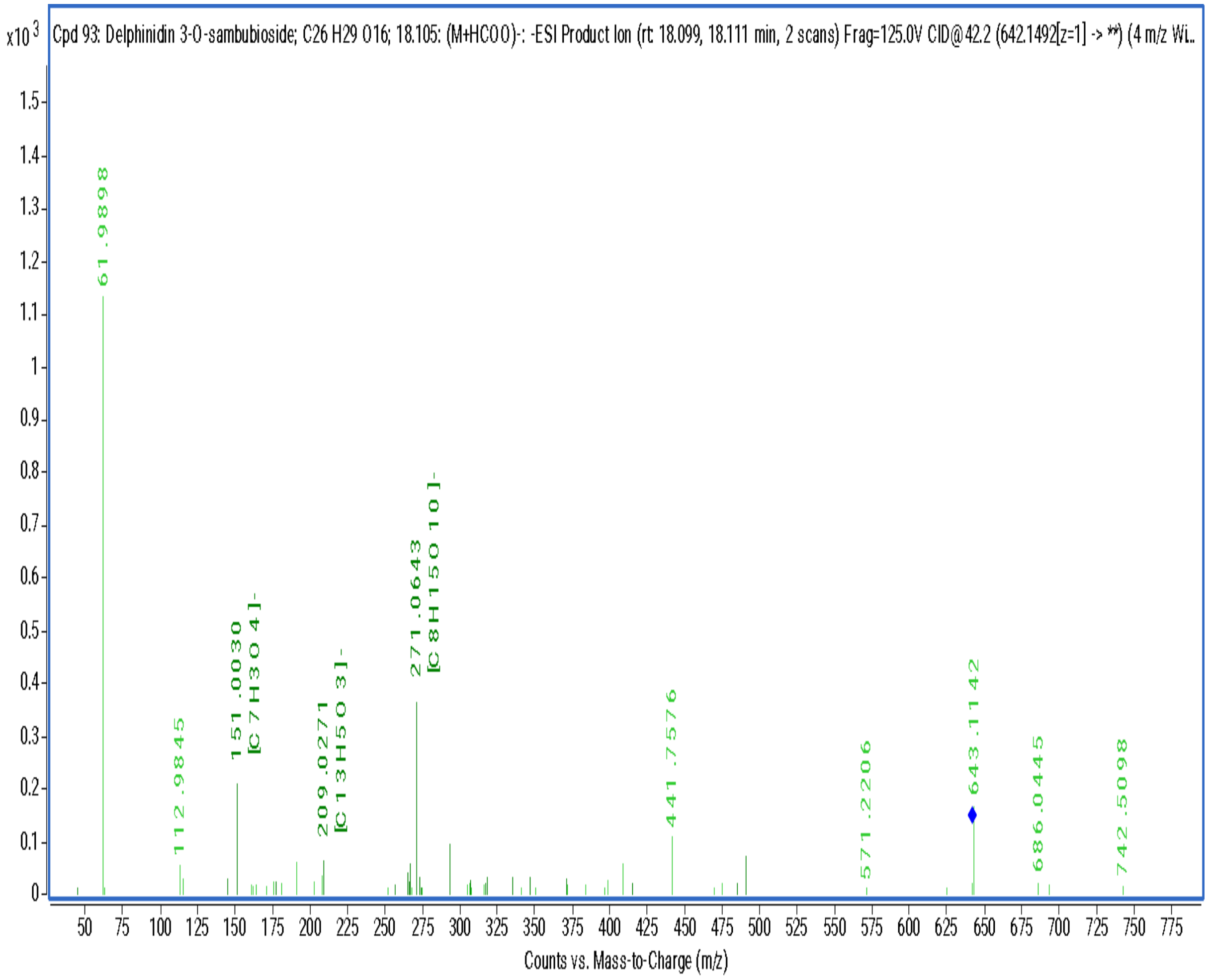

Figure S27. Negative-ion ESI-MS/MS product-ion spectrum of delphinidin 3-O-sambubioside

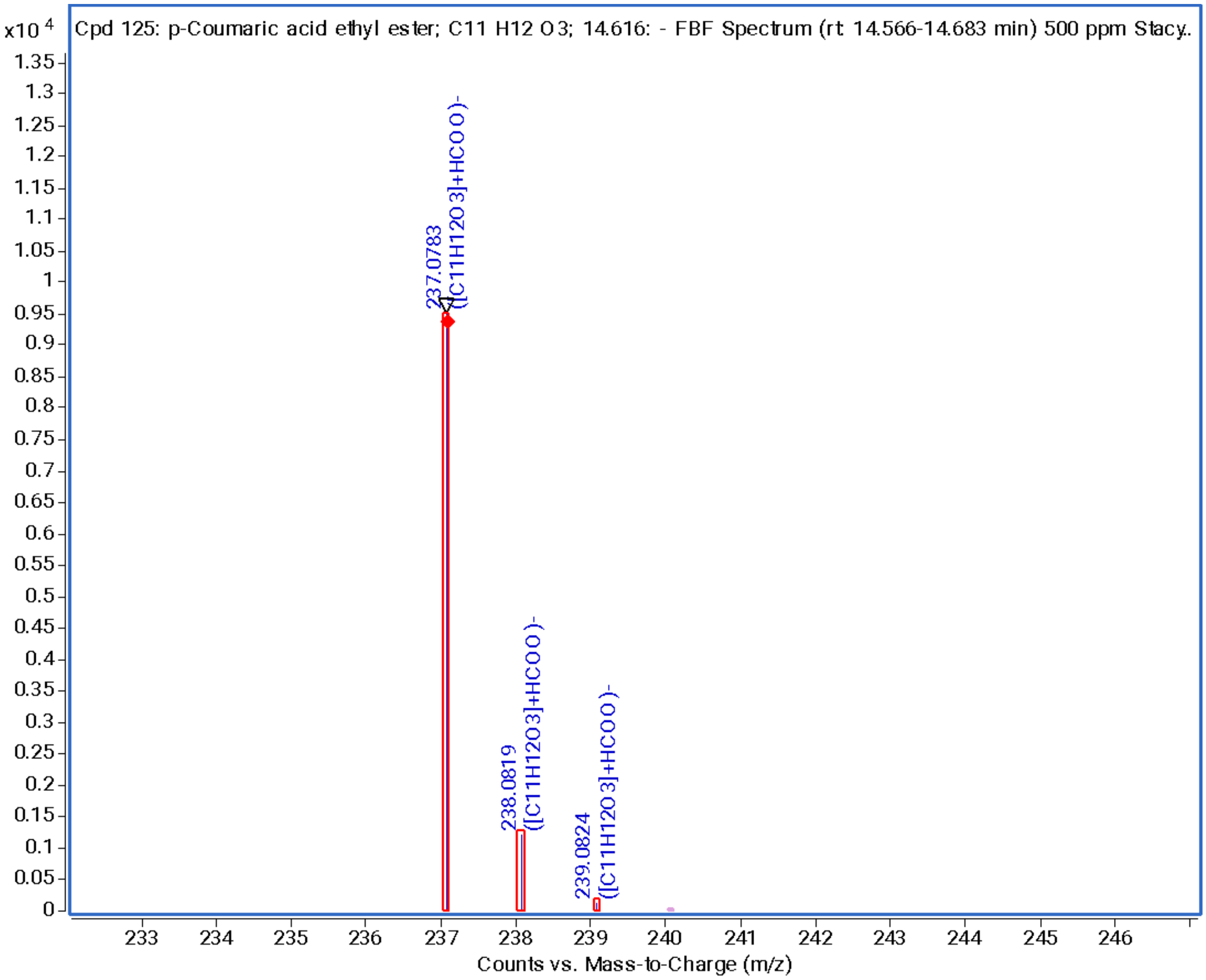

Figure S28. Negative ESI-MS full-scan spectrum of p-coumaric acid ethyl ester

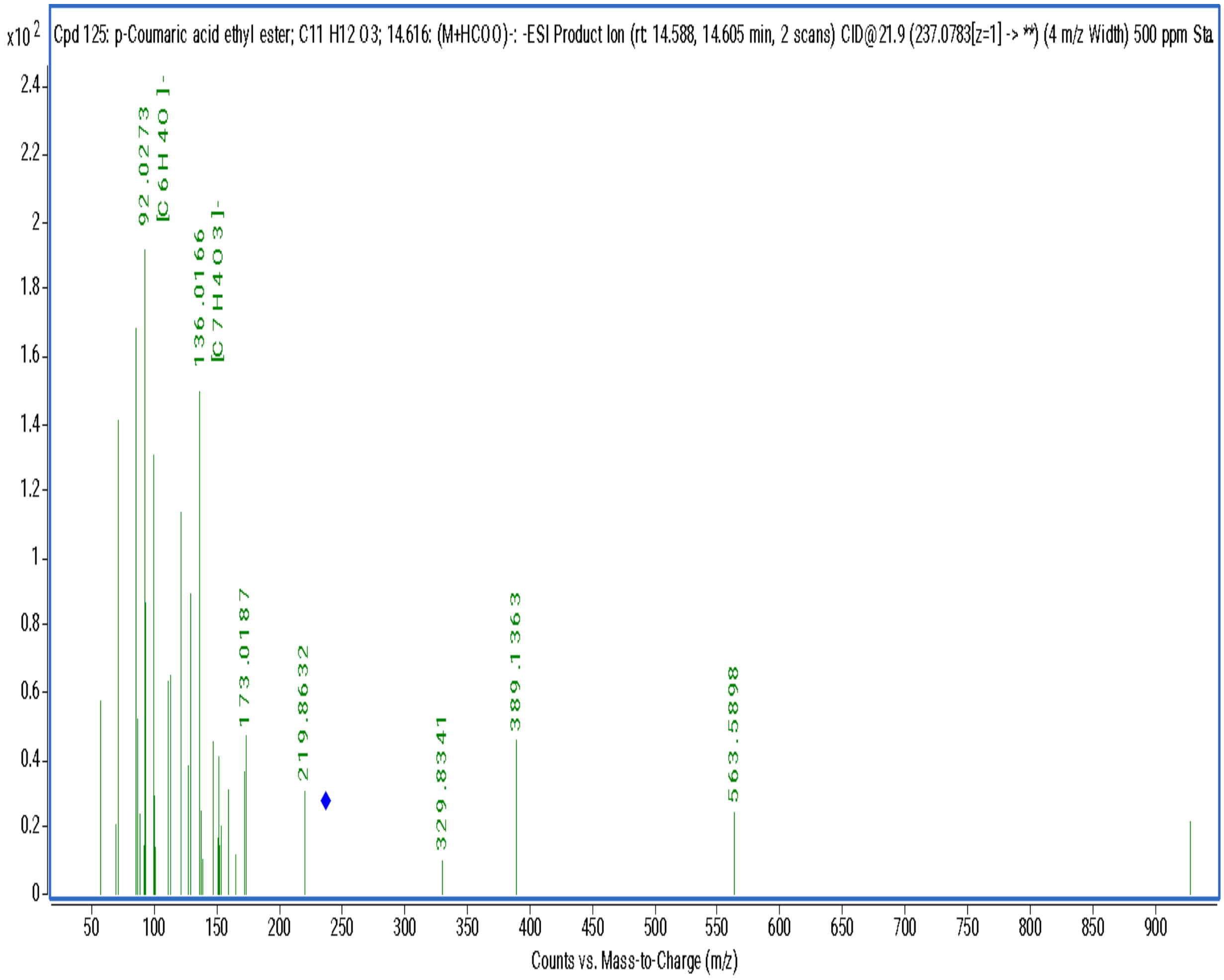

Figure S29. Negative-ion ESI-MS/MS product-ion spectrum of p-coumaric acid ethyl ester

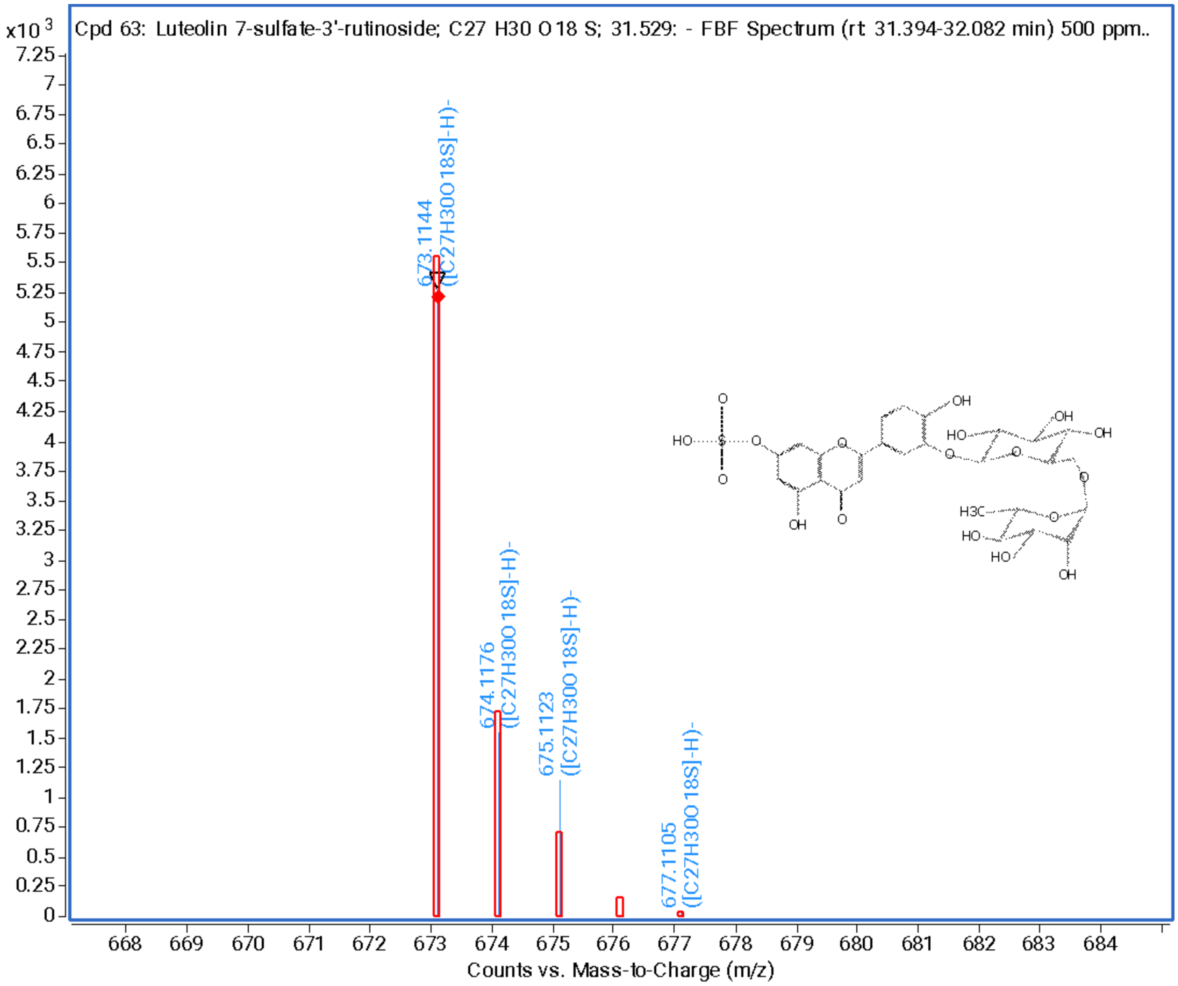

Figure S30. Negative ESI-MS full-scan spectrum of luteolin 7-sulfate-3′-rutinoside

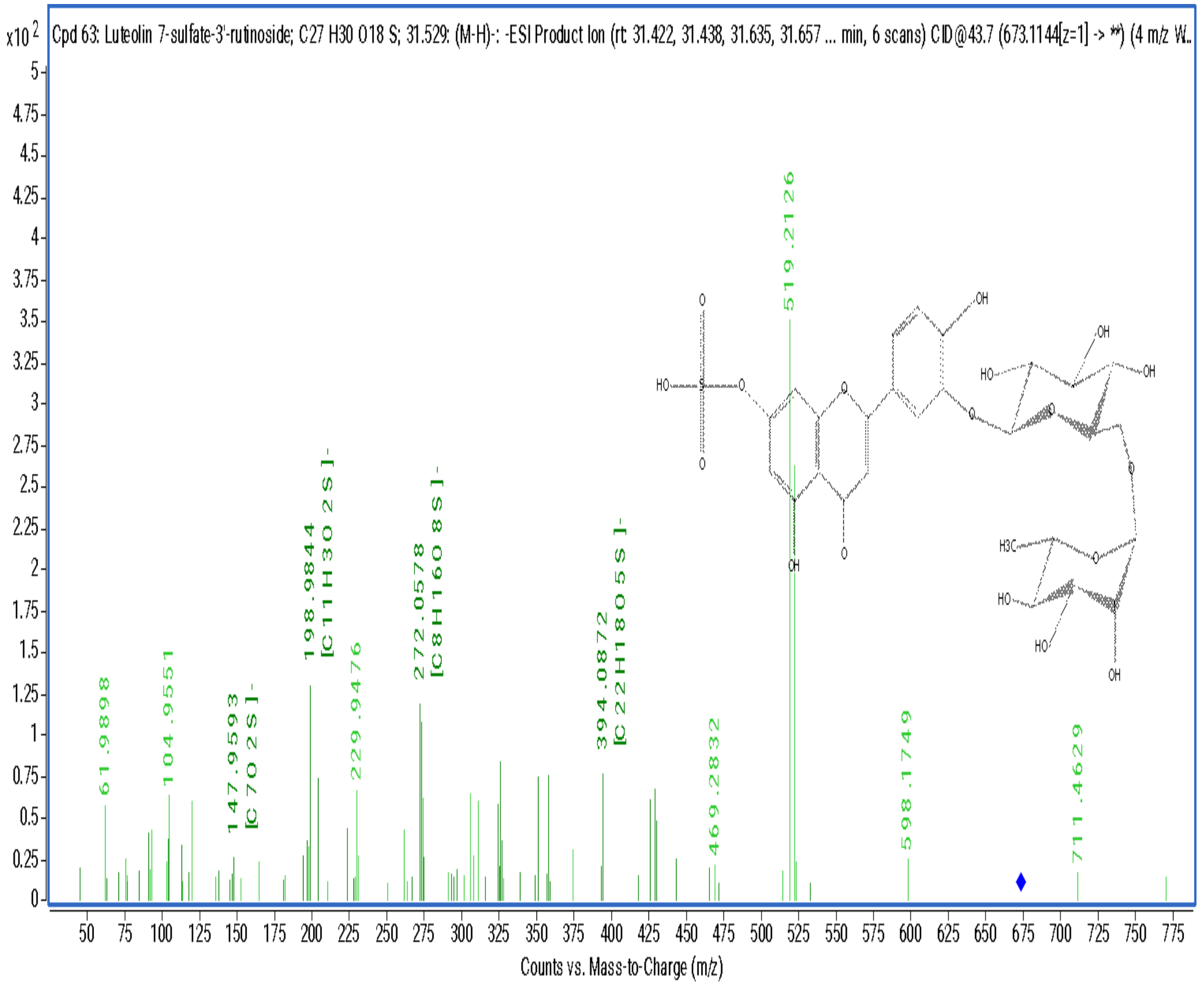

Figure S31. Negative-ion ESI-MS/MS product-ion spectrum of luteolin 7-sulfate-3′-rutinoside

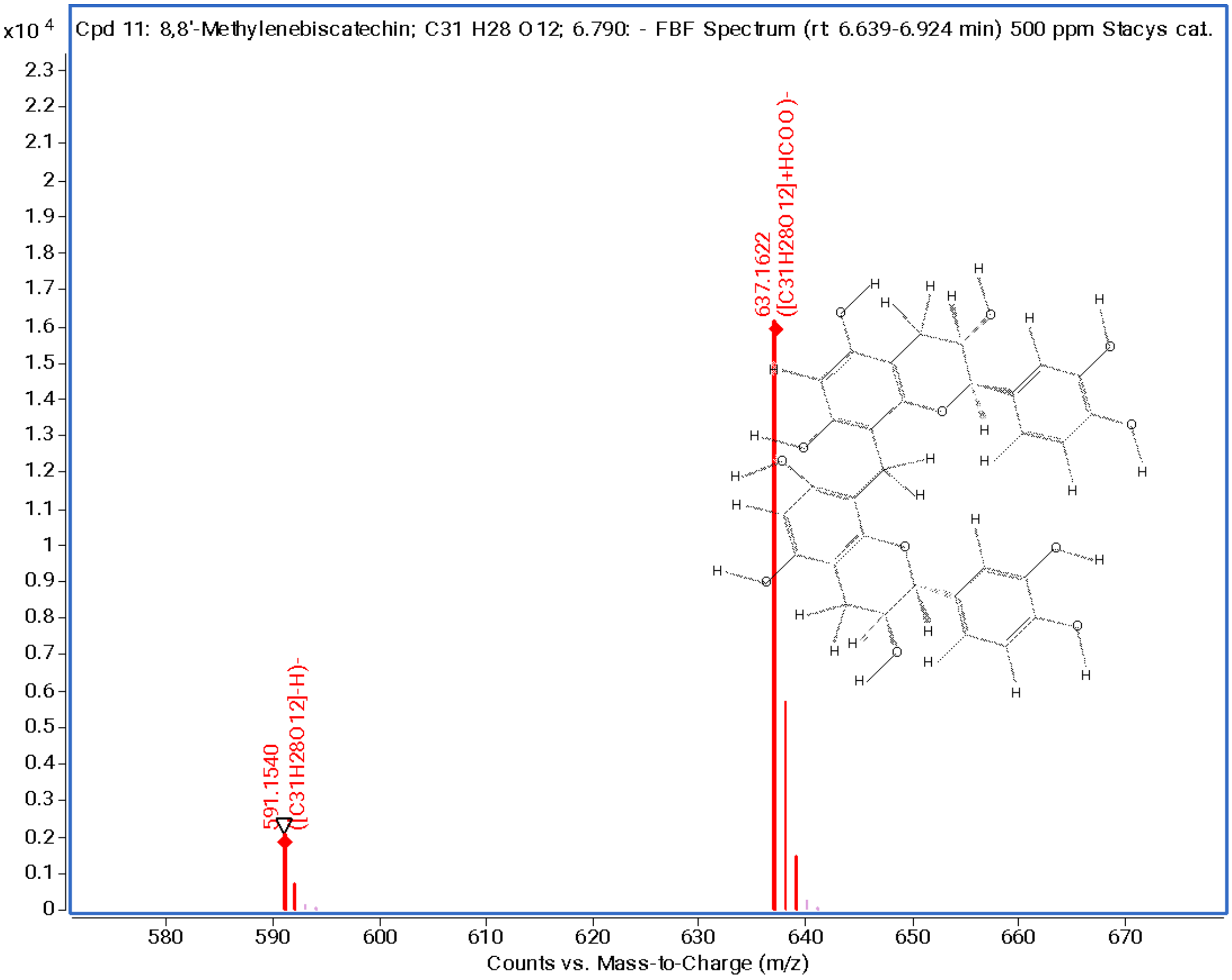

Figure S32. Negative ESI-MS full-scan spectrum of 8,8′-methylenebiscatechin

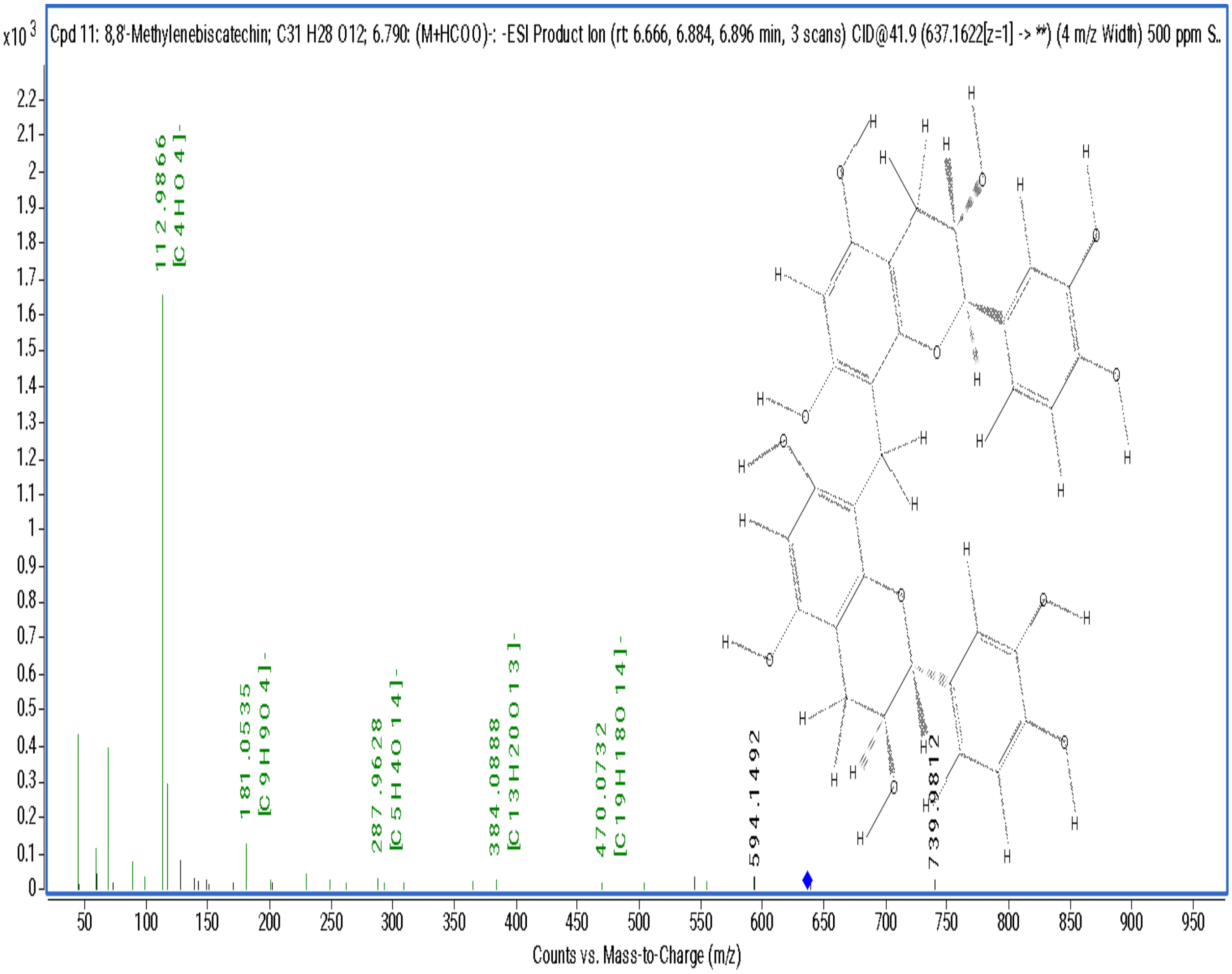

Figure S33. Negative-ion ESI-MS/MS product-ion spectrum of 8,8′-methylenebiscatechin

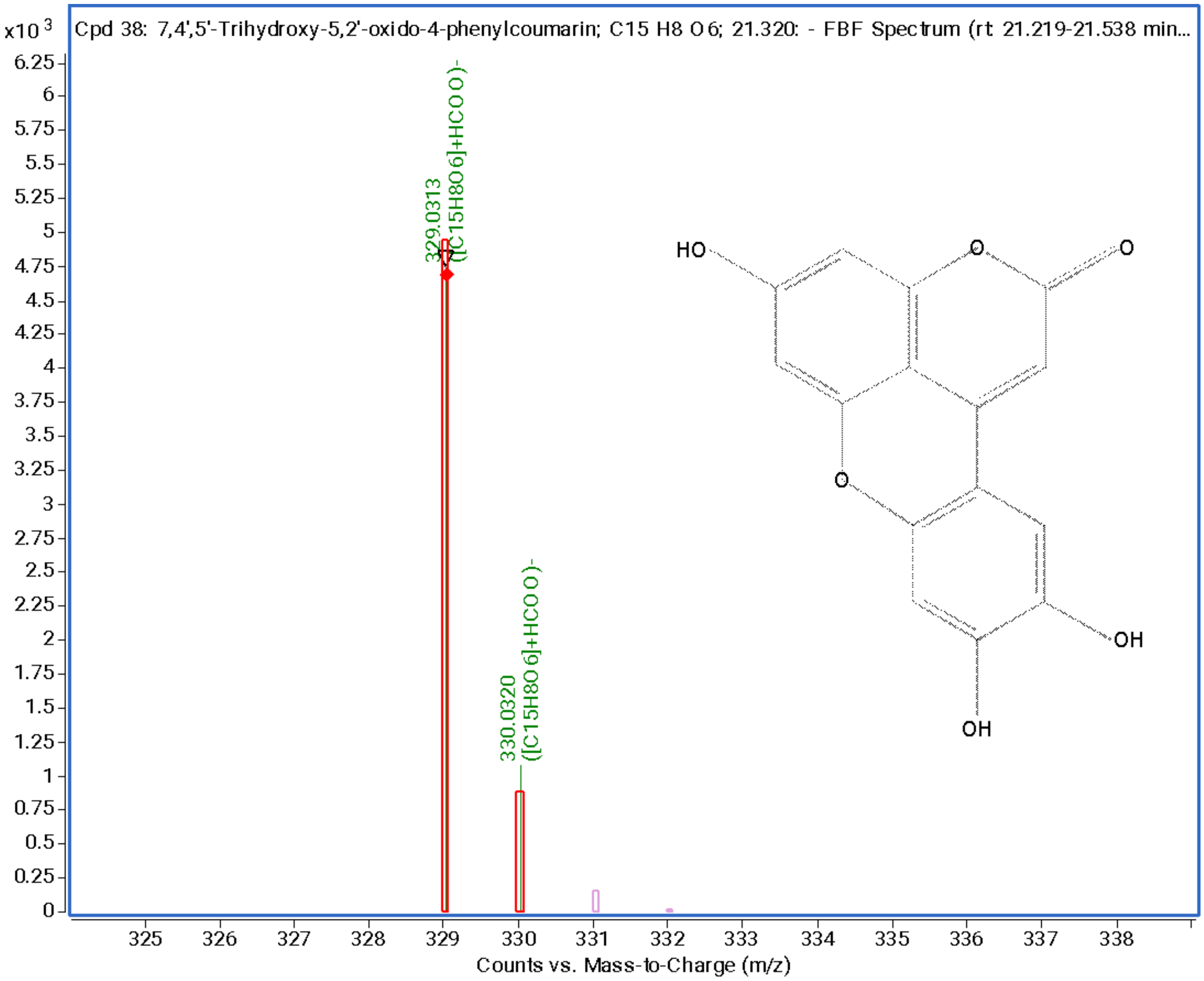

Figure S34. Negative ESI-MS full-scan spectrum of 7,4,5-trihydroxy-5′,2′-oxido-4-phenylcoumarin

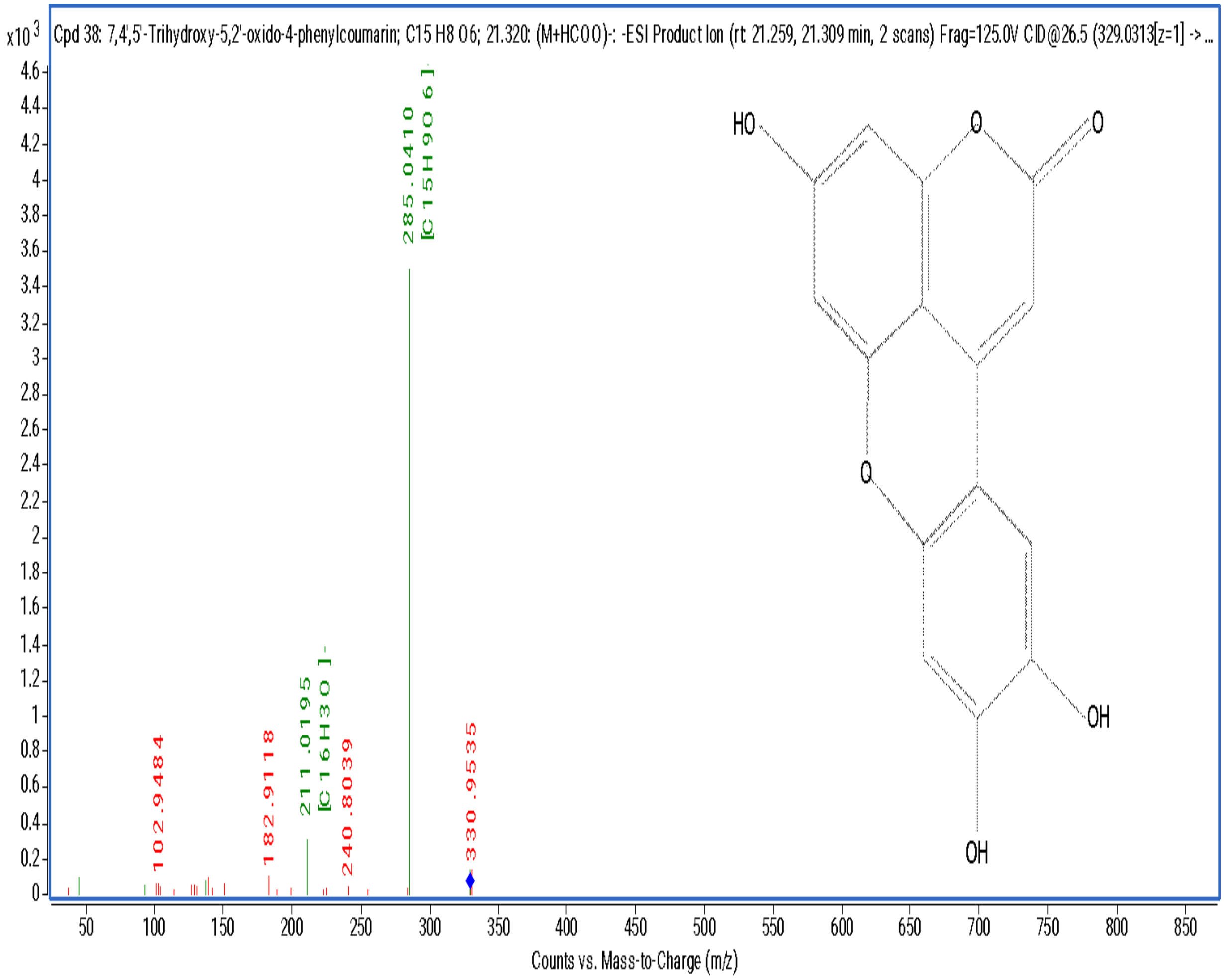

Figure S35. Negative-ion ESI-MS/MS product-ion spectrum of 7,4,5-trihydroxy-5′,2′-oxido-4-phenylcoumarin
